# African swine fever virus ubiquitin-conjugating enzyme pI215L inhibits IFN-I signaling pathway through STAT2 degradation

**DOI:** 10.3389/fmicb.2022.1081035

**Published:** 2023-01-13

**Authors:** Elena Riera, Raquel García-Belmonte, Ricardo Madrid, Daniel Pérez-Núñez, Yolanda Revilla

**Affiliations:** ^1^Microbes in Health and Welfare Department, Centro de Biología Molecular Severo Ochoa, CSIC-UAM, Madrid, Spain; ^2^Bioassays SL, UAM, Madrid, Spain; ^3^Department of Genetics, Physiology and Microbiology, Faculty of Biological Sciences, Biology, UCM, Madrid, Spain

**Keywords:** ASFV, pI215L, ubiquitin-conjugating enzyme, IFN-I, STAT2, proteasomal degradation

## Abstract

African swine fever virus (ASFV) is the causative agent of one of the most lethal diseases affecting domestic pig and wild boar, which is endangering the swine industry due to its rapid expansion. ASFV has developed different mechanisms to evade the host immune response, including inhibition of type I IFN (IFN-I) production and signaling, since IFN-I is a key element in the cellular antiviral response. Here, we report a novel mechanism of evasion of the IFN-I signaling pathway carried out by the ASFV ubiquitin-conjugating enzyme pI215L. Our data showed that pI215L inhibited IFN-stimulated response element (ISRE) activity and the consecutive mRNA induction of the IFN-stimulated genes ISG15 and IFIT1 through the ubiquitination and proteasomal degradation of STAT2. Additionally, by immunofluorescence, co-immunoprecipitation and nucleus-cytoplasm fractionation approaches, we have confirmed the interaction and colocalization of STAT2 and pI215L, in ectopic experiments and during ASFV infection. Moreover, expression of the catalytic mutant (I215L-C85A) did not inhibit the induction of ISG15 and IFIT1, nor the activity of ISRE. Furthermore, we confirmed that STAT2 degradation by pI215L is dependent on its catalytic activity, since expression of the pI215L-C85A mutant did not affect STAT2 levels, compared to the wild-type protein. Yet, our data reveal that the interaction of pI215L with STAT2 does not require the integrity of its catalytic domain since the pI215L-C85A mutant co-immunoprecipitates with STAT2. All these findings reveal, for the first time, the involvement of E2-ubiquitin-conjugating enzyme activity of pI215L in the immune response modulation.

## Introduction

African swine fever (ASF) is a lethal hemorrhagic disease that affects domestic pigs and wild boar, which poses an increasing threat to the pig industry and to the ecosystem of the affected countries, since there are no currently fully safe and effective vaccines available. Although control strategies are being carried out, the disease has spread into many areas of the world, including Russia, Eastern Europe, East Asia, China and recently the Caribbean region (Sánchez-Cordón et al., 2018; Dixon et al., 2020). African swine fever virus (ASFV), the etiological agent of ASF and the only member of the *Asfarviridae* family, is an enveloped nucleocytoplasmic dsDNA virus of huge complexity and size that encodes more than 150 proteins. This includes structural proteins, involved in processes such as virus assembly and entry, and nonessential proteins that have important roles in the evasion of the host-induced antiviral immune responses (Yáñez et al., 1995; Correia et al., 2013; Dixon et al., 2013), including inhibition of type I IFN production (reviewed in García-Belmonte et al., 2022) and apoptosis (Revilla et al., 1997; Hernáez et al., 2004; Banjara et al., 2017).

The innate immune response is triggered by the host’s pattern recognition receptors (PRRs), which identify the evolutionarily conserved pathogen-associated molecular patterns (Kawai and Akira, 2006; Thompson et al., 2011). This pattern recognition leads to the activation of signal transduction pathways to induce type I interferon (IFN-I) production. Once secreted, IFN-I acts both in autocrine and paracrine manners *via* the activation of the JAK/STAT signaling pathway, by binding to its corresponding receptors (IFNAR) and promoting janus kinase (JAK) 1 and tyrosine kinase 2 (TYK2) activation. These activated kinases phosphorylate the signal transducer and activator of transcription (STAT) 1 and STAT2, which allows the recruitment and binding of the interferon regulatory factor 9 (IRF9). IFN-stimulated gene factor (ISGF) 3 complex, composed by STAT1/STAT2/IRF9, translocates into the nucleus and enhance the activity of IFN-stimulated response element (ISRE) promoter, thus supporting the expression of IFN-stimulated genes (ISGs) and the establishment of an antiviral state (Darnell et al., 1994; Meyer and Vinkemeier, 2004; Stark and Darnell, 2012).

The ubiquitination process is one of the most important post-translational modifications in the regulation of cell signaling pathways, being critical in the regulation of the JAK/STAT pathway, by restricting exacerbated IFN-I signaling (Shuai and Liu, 2003; Lurie and Platanias, 2005; Huangfu and Fuchs, 2010). Ubiquitination of STAT proteins, specifically STAT1, was among the first transcription factors ubiquitinated to be reported (Kim and Maniatis, 1996; Zuo et al., 2020), being its levels regulated through the ubiquitin-proteasome pathway. However, confirmation of the factors that make up the ubiquitination complex, as well as the specific mechanisms of STAT1 and STAT2 degradation remain poorly understood. Viruses have hijacked ubiquitination as a process to regulate the IFN-I signaling pathway and thus suppress the antiviral response (review in Viswanathan et al., 2010). STAT2 is described to be a key target of ubiquitin-mediated proteasomal degradation induced by viruses such as human cytomegalovirus (Zimmermann et al., 2005; Trilling et al., 2011), respiratory syncytial virus (Lo et al., 2005; Elliott et al., 2007), paramyxoviruses (Ulane and Horvath, 2002; Ulane et al., 2005) and several flaviviruses such as Dengue virus (Ashour et al., 2009). In fact, our group described that both the virulent Arm/07/CBM/c2 and the attenuated NH/P68 of ASFV strains inhibit the JAK/STAT signaling pathway through proteasomal degradation of STAT2 and caspase-3-dependent cleavage of STAT1 (Riera et al., 2021).

ASFV pI215L is encoded by an ASFV early gene, being present in purified extracellular virions (Hingamp et al., 1995) and is the only known ubiquitin-conjugating enzyme encoded by a virus, sharing a high significant homology with the E2s family of ubiquitin-conjugating enzymes (Hingamp et al., 1992; Rodriguez et al., 1992). pI215L is expressed very early during infection, in the nucleus and the cytoplasm of infected Vero cells. pI215L, as a conjugating enzyme, is able to bind several classes of polyubiquitin chains, being the residue cysteine 85 (C85) essential for its conjugating activity. It has been reported that I215L protein expression is relevant for late gene transcription and also for viral particle production, since its silencing negatively affects both stages of infection (Freitas et al., 2018). pI215L has been implicated in the modulation of the innate immune response by preventing IFN-I production through inhibition of NF-κB and AP1 (Barrado-Gil et al., 2021) and by regulating the cGAS-STING pathway through the inhibition of TBK1 phosphorylation (Huang et al., 2021). Recently, it has been described that I215L modulates IFN-I signaling through degradation of IRF9 *via* the autophagosomal/lysosomal pathway (Li et al., 2022). However, no studies involving ubiquitin-conjugating activity of pI215L as a negative regulator of the IFN-I production or IFN-I signaling have been reported.

In this study we have confirmed the role of ASFV-I215L protein as a negative modulator of the JAK/STAT signaling pathway. We also described that pI215L counteracts the activation of the JAK/STAT pathway through a new mechanism, involving the ubiquitination and degradation of STAT2. Finally, we have determined that STAT2 degradation carried out by pI215L is dependent on its ubiquitin conjugating catalytic activity, being the first time that its ubiquitin conjugating activity is involved in the modulation of the innate immune response.

## Materials and methods

### Cells and viruses

COS-1 (CRL-1650) and Vero cells (CCL-81) from African green monkey kidney were obtained from the American Type Culture Collection (ATCC) and cultivated in DMEM supplemented with 2 mM L-glutamine, 100 U/mL gentamicin, 0.4 mM non-essential amino acids and 5% fetal bovine serum (FBS; Invitrogen Life Technologies). BSRT7 cells, derived from BHK-21 cells (CCL-10, ATCC) stably expressing T7 phage polymerase, were kindly provided by Dr. Adolfo García Sastre (Icahn School of Medicine at Mount Sinai, New York) and grown in DMEM 5% fetal bovine serum (FBS; Invitrogen Life Technologies). Porcine alveolar macrophages (PAM) were obtained by a bronchoalveolar lavage as previously described (Carrascosa et al., 1982) and were cultured in Dulbecco modified Eagle medium (DMEM) supplemented with 2 mM L-glutamine, 0.4 mM nonessential amino acids and 100 U/mL gentamicin with 10% porcine serum. The field virulent strain circulating in Europe Armenia/07/CBM/c2 (Gallardo et al., 2015; Pérez-Núñez et al., 2020) was propagated in PAM and titrated by TCID50 in COS-1 cells and labeling with anti-p32 antibody.

### Antibodies and reagents

In order to determinate pI215L expression and location during ASFV infection, we generated a polyclonal antibody. For this purpose, I215L gene was cloned in a pGEX-4 T plasmid, transformed in *E.coli* BL21 cells and grown in LB medium (10 g tryptone, 5 g yeast extract, 5 g NaCl, pH 7.2) supplemented with ampicillin (100 μg/mL) at 37°C with shaking. When OD600 reached 0.4, I215L-GST protein expression was induced by adding isopropyl-β-D-1-thiogalactopyranoside (IPTG) at a final concentration of 1 mM during an hour and a half. Then, BL21 cells were harvested, centrifuged, resuspended in Tris-Buffered Saline buffer (TBS) supplemented with protease inhibitors and mechanically lysed by passing them through the Homogenizador GEA Niro Soavi. Purification of I215L-GST was carried out using glutathione sepharose 4B GST-tagged protein purification resin (Cytiva). The purification product was treated with thrombin (10 U/mL, Sigma) for 1 hour and a half at room temperature, to cleave and discard GST bound to I215L. The purified sample with thrombin was incubated 1 h at 4°C in the waterwheel with p-AminoBenzamidine-agarose (Sigma), to discard and inactivate thrombin. The concentration of purified recombinant I215L protein was quantified by bicinchoninic acid (BCA; ThermoScientific) and its correct expression and purification was analyzed by Coomassie blue stain. The generation of the polyclonal antibody was carried out through the company Biomedal, which immunized a rabbit with purified recombinant I215L protein (0.5 μg/mL). The serum from the immunization was purified by protein A/G affinity. The specificity of the polyclonal antiserum was tested against infected-cells or I215L-transient expression extracts by Western blotting. The immunoblotting was achieved by the incubation with the primary polyclonal antibody at 1:2,000 in 1% skimmed milk diluted in TBS at 4°C overnight, then labeled with an anti-rabbit immunoglobulin G coupled to peroxidase antibody and finally revealed using ECL Prime Western blotting detection reagent (Amersham Biosciences). Monoclonal rabbit antibody anti-STAT2 (D9J7L), anti-ubiquitin (P37, 58,395), anti-myc tag (9B11, 2,276) and anti-FLAG (2368) antibodies were acquired from Cell Signaling. Monoclonal mouse antibodies anti-STAT2 (B-3, sc-514,193), anti-b-actin (C-4, sc-47,778) and the secondary antibody anti-m-IgGk (BP-HRP, sc-516,102) were purchased from Santa Cruz Biotechnology. Monoclonal antibodies anti-p32 ASFV protein (S-1D8 and S-5C1) were kindly provided by S.Y. Sunwoo. For p72 and pS273R labeling, a house-made polyclonal rabbit antibodies against p72 or pS273R were used. For Western blot experiments, anti-rabbit and anti-mouse immunoglobulin G coupled to peroxidase antibodies and ECL Prime Western blotting detection reagent were purchased from Amersham Biosciences. Secondary antibodies anti-rabbit/Alexa Fluor 488 and anti-mouse/Alexa Fluor 555 from Invitrogen were used in Immunofluorescence. For ISG and ISRE induction, co-localization and co-immunoprecipitation assays, cells were treated with universal type I IFN at the indicated concentrations (PBL; catalog no 11200–1). Cytosine arabinoside (AraC) was employed at a concentration of 40 mg/mL during infection.

### Plasmids and transfection

For I215L-WT transient expression, we cloned I215L-WT gene from Arm/07/CBM/c2 strain (GenBank: LR812933.1) into a pGEM-IRES-myc vector (Ramos and Martínez-Salas, 1999) using In-Fusion HD Cloning technology (Takara). For the generation of the pIRES-I215L-C85A-myc vector, we generated a point mutation in cysteine 85 from I215L corresponding with the catalytic domain in pIRES-I215L-WT-myc vector. For this, the codon coding for cysteine 85 was mutated by PCR to a codon coding for the alanine amino acid. pCAGGS empty vector (chicken β-actin promoter), human-STAT2-FLAG (Miorin et al., 2019) and human-STAT1-FLAG were kindly provided by Dr. Adolfo García Sastre. pCI-His-hUbiquitin vector was acquired from Addgene (#31815). For the transfection protocol, FuGENE HD transfection reagent from Promega was employed, following the manufacturer’s instructions.

### RNA extraction and RT-qPCR

To analyze the mRNA levels of I215L transcribed during infection, 6 × 10^6^ PAM cells were seeded in p60 plates and mock infected or infected with Arm/07/CBM/c2 (2 PFU/cell) in DMEM 10% porcine serum. At the indicated times post-infection, cells were harvested, and the total mRNA was extracted using the RNeasy kit (Qiagen). Retrotranscription of RNA to cDNA was carried out with NZY first-strand cDNA synthesis kit (NZYTech). For analysis of ISGs mRNA induction, COS-1 cells were seeded in M6 wells and transfected with pCAGGs empty vector (2.5 μg/1×10^6^ cells), with pIRES-I215L-WT-myc or with pIRES-I215L-C85A-myc (0.5 or 2.5 μg/1×10^6^ cells). Twenty-four hours post-transfection, cells were stimulated or not with IFN-I (500 U/mL) for 8 h, after which they were harvested and processed for mRNA extraction as previously specified. Viral I215L, ISG15, IFIT1, viral p32 (CP204L), and viral p72 (B646L) mRNA levels were evaluated by quantitative reverse transcription PCR (RT-qPCR) with CFX-384 (BioRad) detection system. Gene expression levels were normalized to the housekeeping gene 18S rRNA and these values where then relativized to the mock infected sample or to the pCAGGs empty vector transfected and untreated sample. The primers employed for the RT-qPCR experiments were: 5′- AGGGAATGACTTGGTCTCCG-3′ and 5′-CGG TAG CTT TTA GCT GCA TCC-3′ for viral I215L detection; 5′-GGTGCAAAGCTTCAGAGACC-3′ and 5′-GTCAGCCAGACCTCATAGGC-3′ for ISG15 detection; 5′- GCTGCTGTTTAGCTCCCTTA-3′ and 5′- TGGTTGCTGTGAATTAGGCA-3′ for IFIT1 detection; 5′-GCAGGAAAGGGCAACAATAA-30 and 50-GAGGGTGTCCGTTGTCAGTT-3′ for STAT2 detection; 5’-AAAAATGATAATGAAACCAATGAATG-3′ and 5′-ATGAGGGCTCTTGCTCAAAC-3′ for viral p32 detection; 5′-TGCATAGGAGAGGGCCACTA-3′ and 5′-CCAGGGGATAAAATGACTGG-3′ for viral p72 detection and 5′-GGCCCGAGGTTATCTAG AGTC-3′ and 5′-TCAAAACCAACCCGGTCA-3′ for 18S detection.

### Western blots

At the indicated times post-infection or post-transfection, cells were harvested, washed with Phosphate-buffered saline (PBS), and lysed with radioimmunoprecipitation assay (RIPA) buffer (50 mM TrisHCl pH 7.4, 150 mM NaCl, 1% Triton, 0.5% Deoxycholate, SDS 0.1%), supplemented with protease and phosphatase cocktail inhibitors (Roche). Sample lysates were centrifuged at 13,000 rpm for 10 min at 4°C and the supernatants were collected. Protein concentration was determined employing a Pierce BCA Protein Assay kit (ThermoScientific). Equal protein amounts were resolved by sodium dodecyl sulfate polyacrylamide gel electrophoresis (SDS-PAGE) and then transferred to Immobilon-P membrane (Millipore). Membranes containing the transferred proteins were incubated with the following antibodies: anti-STAT2 (1: 1,000), anti-pSTAT2 (Tyr690, D3P2P), anti-I215L (1: 2,000), anti-ubiquitin (1: 1,000), anti-myc (1: 1,000), anti-FLAG (1: 4,000), anti-p72 (1: 2,000), anti-p32 (S-1D8, 1: 6,000), anti-S273R (1: 2,000), anti-actin (1: 6,000) and anti-lamin B1 (1: 2,000). Incubation with the primary antibody was performed overnight at 4°C. After incubation, membranes were washed with TBS and then exposed to specific peroxidase conjugated secondary antibodies: anti-mouse (1: 2,000), anti-rabbit (1: 5,000) or anti-m-IgGk secondary antibody (1: 1,000) immunoglobulin G coupled to peroxidase. Protein bands were visualized by chemiluminescence detection using ECL Prime (Amersham Biosciences).

### Luciferase reporter assays

HEK-293 cells were seeded in M24 plates and co-transfected with pCAGGs empty vector (3 μg/1×10^6^ cells), pIRES-I215L-WT-myc or pIRES-I215L-C85A-myc (0.3, 1 or 3 μg/1 × 10^6^ cells), pISRE-firefly-luc (100 μg/1 × 10^6^ cells) and Renilla luciferase reporter construct pRLTK (50 μg/1 × 10^6^ cells). At 24 h post-transfection, cells were either untreated or treated with 500 U/mL of universal type I IFN (PBL). At 16 hpi, cells were collected and processed according to the manufacturer indications and the luciferase activity was measured using the Luc-PairTM DuoLuciferase HS Assay Kit (GeneCopoeia) and carried out with FLUOstar OPTIMA reader (BMG LabTech).

### Immunoprecipitation assay

For the analysis of I215L-WT/I215L-C85A interaction with STAT2 or STAT1 under ectopic conditions, BSRT7 cells were seeded in p100 plates and co-transfected with hSTAT2-FLAG or hSTAT1-FLAG (0.4 μg/1 × 10^6^ cells) and with pCAGGs empty vector, pIRES-I215L-WT-myc or pIRES-I215L-C85A-myc (2 μg/1 × 10^6^ cells). 24 h post-transfection, cells were treated with IFN-I (500 U/mL) for 2 h, collected and lysed following the Pierce™ Classic Magnetic IP/Co-IP Kit (ThermoFisher) manufacturer instructions. A portion of the lysates (Input) was collected, and the rest was employed to the immunoprecipitation (IP). A/G magnetics beads bound to anti-myc tag antibody (Thermo Scientific™, No Catalog 88842) for myc immunoprecipitation or beads washed and bound to STAT2 antibody (1:50 dilution) for STAT2 immunoprecipitation were used. A crosslink of the beads attached to the antibody was made to promote an irreversible binding, following the manufacturer instructions. Subsequently, the rest of the lysates were incubated with the beads bound to the antibody overnight at 4°C under rolling agitation. Beads were then washed, and the bound proteins were eluted with Kit’s elution buffer. Input and immunoprecipitated samples were loaded into SDS-PAGE followed by immunoblot analysis. Antibodies against STAT2, myc and actin were employed.

For the study of ubiquitinated STAT2 in the presence of I215L-WT or I215L-C85A, Vero or COS-1 cells were used. Cells were seeded in p100 plates and co-transfected with hSTAT2-FLAG (0.4 μg/1 × 10^6^ cells), pCI-His-hUbiquitin vector (1 μg/1 × 10^6^ cells; Addgene, #31815) and with pIRES-I215L-WT-myc or pIRES-I215L-C85A-myc (2 μg/1 × 10^6^ cells). STAT2 immunoprecipitation was performed as explained above. Input and immunoprecipitated samples were loaded into SDS-PAGE followed by immunoblot analysis using antibodies against STAT2, ubiquitin, myc and actin were employed.

### Pull down

BL21 bacteria expressing GST or I215L-GST were lysed in buffer containing 25 mM Tris (pH 7.6), 150 mM NaCl, 5 mM EDTA, 2 mM MgCl_2_, 1 mM DTT, PMSF 1 μM and protease inhibitors [aprotinin (10 mg/mL), pepstatin (1 mg/m), leupeptin (5 mg/mL)]. The bacteria were then sonicated and lysed with lysozyme (0.3 mg/ml) and NP40 (0.5%) for 1 h at 4°C under agitation. After this time, lysozyme was added again at the same concentration and incubated for 1 h at 4°C. The bacterial lysates were then centrifuged for 10 min at 12,000 rpm at 4°C. The supernatant was collected and incubated with glutathione high-capacity magnetic agarose beads (Sigma) overnight. After incubation time, the beads were washed, and the expression of GST and bead-bound I215L-GST was confirmed by electrophoresis and Coomassie blue staining. Cellular extracts from porcine alveolar macrophages infected with Arm/07/CBM/c2 for 16 h were lysed in the buffer containing 50 mM Tris (pH 7.6), 150 mM NaCl, 2 mM EDTA, 1% Triton X-100 and protease and phosphatase inhibitors. After 1 h of lysis at 4°C under agitation, the lysates were centrifuged for 10 min at 13,000 rpm. The supernatants were collected, from which part was removed for Input and the remainder was incubated with the agarose beads bound to GST or I215L-GST overnight. Next day, the unbound fractions were discarded, the beads were washed, and elution of the samples were carried out at 95°C for 10 min. Finally, proteins from the PAMs infected extracts bound to GST or I215L-GST were analyzed by Western blot.

### Immunofluorescence

Analysis of colocalization between pI215L-myc and STAT2 by immunofluorescence was performed in Vero cells. Vero cells were transfected with pCAGGs empty vector or pIRES-I215L-WT-myc (2 μg/1 × 10^6^ cells) expression plasmids. After 24 h, cells were stimulated with IFN-I (250 U/mL) during 1 h. After stimulation, cells were fixed with 4% paraformaldehyde for 20 min at room temperature (RT) and 10 min with methanol at −20°C. Then, cells were blocked with 5% porcine serum and 0.3% Triton X-100 in PBS for 1 h. Cells were stained with the primary antibodies rabbit anti-STAT2 (1: 150) and mouse anti-myc (1: 300) diluted in PBS 1% BSA 0.3% Triton X-100 overnight at 4°C. Cells were washed with PBS and labeled with the fluorescent conjugated secondary antibodies anti-rabbit/Alexa Fluor 488 (1: 500) or anti-mouse/Alexa Fluor 555 (1: 500), diluted in 1% BSA, 0.3% Triton X-100 in PBS for 1 h. Then, cells were washed and the coverslips were mounted with DAPI Fluoromount-G (SouthernBiotech). Immunofluorescence to confirm pI215L-STAT2 colocalization and pI215L distribution during infection, PAM cells were seeded on coverslips and infected with Arm/07/CBM/c2 (1 PFU/cell) for 6 and 16 hpi. At 5 or 15 hpi, cells were stimulated or not with IFN-I (250 U/mL) for 1 hour. Cells were processed as above and labeled with mouse anti-STAT2 (1:50) and/or rabbit anti-pI215L (1:100) antibodies. Images were taken by using Nikon A1R *+ in vivo* coupled to an inverted Eclipse TiE microscope (Nikon) with a 60x oil immersion objective lens. Images were imported into ImageJ software for analysis.

### Cellular fractionation

PAM were seeded in p60 plates (6 × 10^6^ cells/plate) and mock infected or infected with the virulent strain Arm/07/CBM/c2 (1 PFU/cell). At 15 h post-infection (hpi), cells were then treated or not with IFN-I (500 U/mL) for 1 h. The whole cell extract (WCE), cytoplasmic fraction (S2) and chromatin fraction (P3) were isolated as described previously (Méndez and Stillman, 2000; García-Belmonte et al., 2019; Riera et al., 2021). Briefly, cells were resuspended in the buffer composed by 10 mM HEPES (pH 7.9), 1.5 mM MgCl_2_, 0.34 M sucrose, 10 mM KCl, 10% glycerol, 0.1 mM phenylmethylsulfonyl fluoride (PMSF), 1 mM dithiothreitol (DTT), protease and phosphatase inhibitors (Roche). This lysate corresponded to the WCE fraction. Then, Triton X-100 was added followed by incubation for 5 min on ice and the nuclei were centrifuged for 4 min at 3,600 rpm at 4°C. The supernatant obtained after centrifugation was collected and centrifuged at 14,000 rpm for 15 min at 4°C, corresponding to the cytoplasmic fraction. The pellet obtained in the first centrifugation was washed with the previous buffer and suspended in a buffer containing 3 mM EDTA, 0.2 mM EGTA, 1 mM DTT, 0.1 mM PMSF and protease and phosphatase inhibitors (Roche). After 30 min of incubation on ice, the pellet lysate was centrifuged for 4 min at 4,000 rpm at 4°C. The pellet corresponding to the chromatin fraction was washed and re-suspended in loading buffer. Finally, the extracts from the different fractions were analyzed by Western blot.

## Results

### ASFV protein I215L counteracts IFN-I signaling pathway

**Figure 1:**
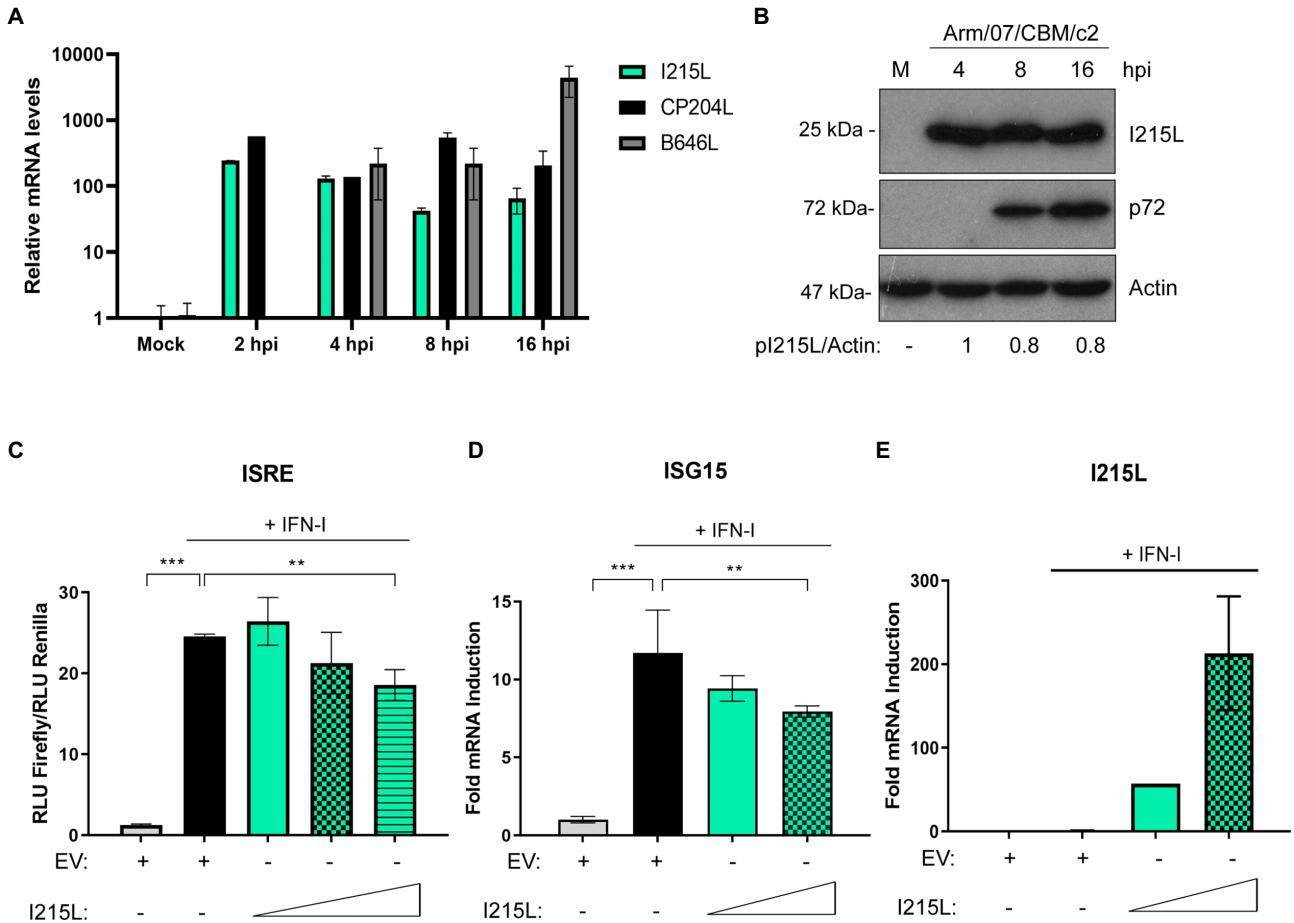
ASFV protein I215L inhibits IFN-I signaling pathway. **(A)** I215L transcripts were detected in mock-infected or infected PAM cells by qRT-PCR. PAMs were mock-infected or infected with the virulent Arm/07/CBM/c2 ASFV strain (2 PFU/cell) and collected at 2, 4, 8, or 16 h post-infection for mRNA extraction. As a control of an early-late and late viral gene expression, CP204L and B646L mRNA were measured, respectively. **(B)** I215L gene encodes for an early protein detected from 4 hpi by Western blot. Arm/07/CBM/c2 infected PAMs (2 PFU/cell) were collected at 4, 8, or 16 hpi, lysed in RIPA buffer and analyzed by Western blot. Antibodies against ASFV-pI215L, p72 (ASFV late protein) and actin were used. pI215L levels were quantified according with their actin levels and relativized to the 4 hpi sample by using ImageJ. **(C)** ISRE-luciferase reporter experiment was performed in HEK-293 cells transfected with empty vector control (EV) or with increasing concentrations of I215L (0.3, 1, or 3 μg/1 × 10^6^ cells) in absence or in presence of IFN-I (500 U/mL). Graph represents the means of the Firefly luciferase RLU (Relative Luminiscence Units) values divided by its Renilla luciferase RLU values from biological triplicates. Obtained values were relativized against the untreated EV transfected sample. **(D)** ISG15 mRNA levels were analyzed in COS-1 cells transfected with EV or increasing concentrations of pI215L (0.5 or 2.5 μg/1 × 10^6^ cells) in absence or in presence of IFN-I by qRT-PCR. **(E)** I215L mRNA levels were amplified by qRT-PCR in the same conditions than **(D)** to verify the efficient I215L transcription in transfected HEK-293 cells. All data reflect mean ± SEM (*n* = 3). Data were statistically analyzed by using a Student *t*-test (**p < 0.01; ***p < 0.001).

We have recently described that both the virulent Arm/07/CBM/c2 and the attenuated NH/P68 ASFV strains modulate the IFN-I signaling pathway by degrading STAT1 and STAT2 (Riera et al., 2021) to evade the innate immune response. Furthermore, we confirmed that the viral factor(s) involved in the inhibition of that pathway should be an early viral factor, as STAT1 and STAT2 degradation occurred in the presence of the inhibitor of the viral DNA replication cytosine arabinoside (AraC), which prevent the expression of late ASFV genes (Rodríguez and Salas, 2013). Therefore, we set out to identify the factor(s) involved in the evasion of JAK/STAT signaling pathway by studying the ASFV E2-ubiquitin conjugating enzyme pI215L, since it has already been described as an inhibitor of IFN-I production to evade the antiviral response (Barrado-Gil et al., 2021; Huang et al., 2021). First, we analyzed both the pI215L mRNA (Figure 1A) and protein levels (Figure 1B; Supplementary Figure 1) in Arm/07/CBM/c2-infected porcine alveolar macrophages (PAM), at different times post-infection. We detected pI215L gene transcription from 2 hpi, with a slight drop between 4 and 8 hpi, that then increased at 16 hpi (Figure 1A). Regarding protein levels, pI215L was detected from 4 hpi to 24 hpi, with a subtle decrease between 8 and 16 hpi, according with the mRNA levels (Figure 1B; Supplementary Figure 1). In addition, we confirmed the early expression of pI215L with AraC treatment by Western blot (Supplementary Figure 1). These data are in line with that observed for pI215L from other ASFV isolates (Freitas et al., 2018) confirming that pI215L is an early-late protein in the viral cycle.

To assess the role of pI215L on the JAK/STAT pathway, we performed an ISRE-dependent luciferase reporter assay. For this purpose, HEK-293 cells were co-transfected with empty vector (EV) or with increasing concentrations of pI215L and with ISRE-luciferase reporter plasmids and then stimulated or not with universal type I IFN. As shown in Figure 1C, pI215L expression negatively affected ISRE reporter activation in response to IFN-I compared to EV-transfected and IFN-I-stimulated samples, showing a clear dose-dependent effect. To confirm that pI215L inhibits IFN-I-mediated signaling, the mRNA levels of ISG15 were analyzed by qRT-PCR in COS-1 cells (Figure 1D). ISG15 mRNA was induced after IFN-I treatment in EV transfected cells, whereas it was inhibited by pI215L transient expression. As a control of pI215L ectopic expression at increasing concentrations, pI215L mRNA levels were also measured (Figure 1E).

### pI215L interacts with STAT2 and both molecules colocalize in the nucleus in IFN-I stimulated cells

**Figure 2:**
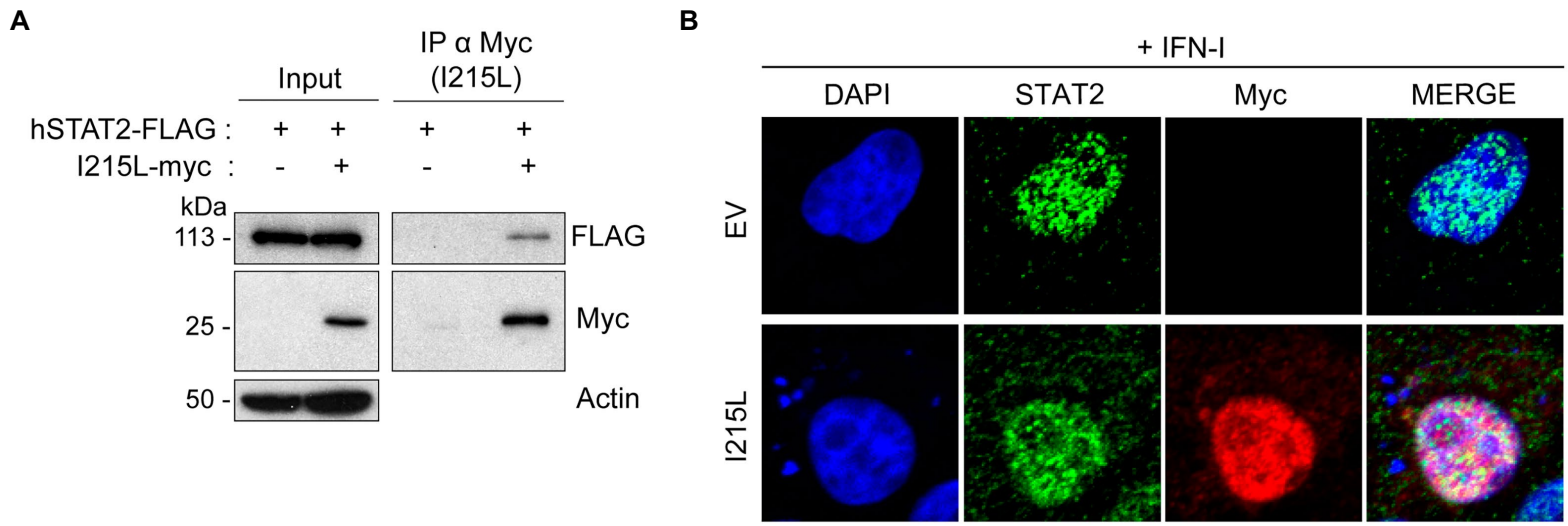
pI215L colocalizes with STAT2 at the nucleus. **(A)** hSTAT2-FLAG interacts with pI215L in BSRT7 cells. BSRT7 cells were co-transfected with hSTAT2-FLAG (0.4 μg/1 × 10^6^ cells) and with pIRES-I215L-myc (2 μg/1 × 10^6^ cells). 24 h post-transfection, cells were stimulated with Universal Type I IFN (500 U/mL) for 1 h. Cells were then collected and processed for immunoprecipitation with an anti-myc antibody and analyzed by Western blot labeling with antibodies against FLAG to detect STAT2, against myc to detect pI215L and against actin. **(B)** pI215L colocalizes with STAT2 in the cell nucleus. Vero cells were transfected with EV or pIRES-I215L-myc (2 μg/1 × 10^6^ cells) expression plasmids. After 24 h, cells were stimulated with IFN-I (250 U/mL) for 1 h. After treatment, cells were fixed and stained with DAPI (blue), anti-STAT2 (green) and anti-myc (I215L; red) antibodies and examined by a confocal microscope. Merged images of the different channels are also shown.

The data shown above suggest that the ASFV-I215L protein may be involved in the inhibition of the JAK/STAT signaling pathway, thus we next approached the identification of the specific molecular mechanism implicated in the modulation of this pathway. Since pI215L has been described to be a ubiquitin-conjugating enzyme (Hingamp et al., 1992; Rodriguez et al., 1992), and we have previously demonstrated that STAT2 is degraded during ASFV infection by the proteasomal system (Riera et al., 2021), we wondered whether pI215L might be directly involved in this degradation. Therefore, we first analyzed the interaction between pI215L and STAT2 during ectopic expression of pI215L (pIRES-I215L-myc) in BSRT7 cells co-transfected with hSTAT2-FLAG after IFN-I treatment (Figure 2A). Immunoprecipitation of pI215L with an anti-myc antibody showed an interaction with hSTAT2-FLAG upon immunoblotting with an anti-FLAG antibody in a Western blot assay. Furthermore, we have used Vero cells for immunofluorescence experiments, as this cell line has been shown to efficiently respond to IFN-I stimulation (Grant et al., 2016 or Ashour et al., 2009). These results indicated that in IFN-I stimulated Vero cells pI215L and STAT2 colocalized in the nucleus (Figure 2B). In addition, the interaction of pI215L with the other component of the ISGF3 complex, STAT1, was analyzed (Supplementary Figure 2). Immunoprecipitation was performed under the same conditions as Figure 2A. The results showed that STAT1 coimmunoprecipitated with pI215L, suggesting that pI215L-STAT2 interaction occurs when the active heterodimer is conformed.

These results explain that STAT2 levels are not affected by pI215L prior to its translocation to the nucleus and suggest a role for the viral protein at this cell compartment.

### pI215L expression induces STAT2 ubiquitination and degradation

**Figure 3:**
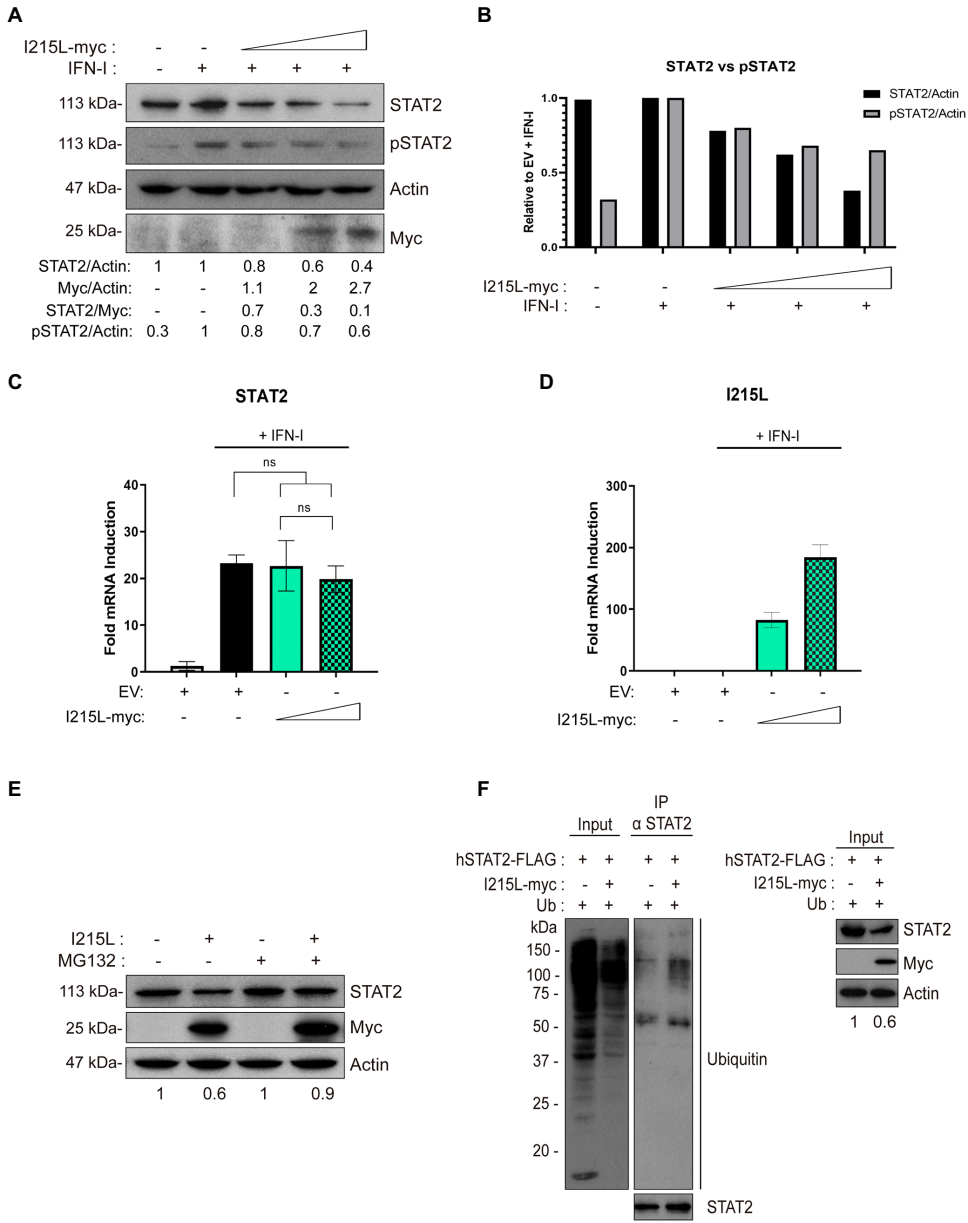
pI215L induces STAT2 ubiquitination and degradation. **(A)** STAT2 and pSTAT2 levels were analyzed in presence of increasing concentrations of pIRES-I215L-myc (0.25, 1, or 2.5 μg/1 × 10^6^ cells) or EV (2.5 μg/1 × 10^6^ cells) in HEK-293 by Western blot assay. Antibodies against STAT2, pSTAT2, myc and actin were employed. STAT2, pSTAT2, and pI215L-myc levels were quantified according with their actin levels and relativized with the EV control using ImageJ. The dose effect of pI215L-myc on STAT2 was quantified as STAT2/Myc. **(B)** Graphical representation of STAT2 versus pSTAT2 levels from the quantification of the Western blot analyzed samples corresponding to **(A)**. **(C)** STAT2 mRNA levels were analyzed in COS-1 cells transfected with EV (2.5 μg/1 × 10^6^ cells) or increasing concentrations of pIRES-I215L (0.5 or 2.5 μg/1 × 10^6^ cells) in absence or in presence of IFN-I by qRT-PCR. **(D)** I215L mRNA levels were amplified by qRT-PCR in the same conditions than **(C)** to verify the correct transcription of I215L in COS-1 transfected cells. All data reflect mean ± SEM (*n* = 3). Data were statistically analyzed by using a Student t test (ns, not significant). **(E)** STAT2 levels were analyzed in presence or absence of the proteasome inhibitor MG132 (10 μM) in pIRES-I215L-myc or EV HEK-293 T transfected cells (2 μg/1 × 10^6^ cells) by Western blot assay. Antibodies against STAT2, myc and actin were employed. STAT2 levels were quantified according with their actin levels and relativized with the corresponding EV control using ImageJ. **(F)** Immunoprecipitation of STAT2 in COS-1 cells transfected with EV or pIRES-I215L-myc. COS-1 cells were co-transfected with pCI-His-hUbiquitin (1 μg/1 × 10^6^ cells), hSTAT2-FLAG (0.4 μg/1 × 10^6^ cells) and either EV or pIRES-I215L-myc plasmids (2 μg/1 × 10^6^ cells). At 24 h post-transfection, cells were treated with IFN-I (500 U/mL) for 2 h. Cells were collected and lysed for STAT2 immunoprecipitation assay with A/G magnetic beads. Western blot labeling with anti-ubiquitin, anti-STAT2, anti-myc and anti-actin antibodies is shown.

We next decided to assess whether pI215L was involved in STAT2 degradation, and to determine if it was proteasome-dependent. For this purpose, we analyzed total STAT2 levels in HEK-293 (Figure 3A), as well as in COS-1 and Vero cells (Supplementary Figure 3) transfected with increased amounts of the pIRES-I215L-myc construct. As shown in Figure 3A and Supplementary Figure 3, the endogenous STAT2 levels were reduced by pI215L expression in a dose-dependent manner, being this reduction more efficient in HEK-293 cells than in monkey cells. In agreement, STAT2 phosphorylation was also affected by pIRES-I215L-myc expression, displaying a decreasing tendency as the dose of I215L-myc was increased, normalized to STAT2 total levels (Figures 3A, B). To further determine the mechanisms by which STAT2 levels decrease as pI215L levels increase, we first quantified the amount of STAT2 mRNA in COS-1 transfected cells with the pIRES-I215L-myc in absence or presence of IFN-I. As shown, none of the concentrations (1 or 5 μg) of pIRES-I215L-myc affected STAT2 mRNA levels compared to control EV-transfected cells (Figures 3C, D). In summary, these results indicated that the downregulation of STAT2 levels by pI215L expression does not involve an effect on STAT2 transcription. Thus, we next investigated if the effect of pI215L expression on STAT2 involves a degradation mechanism. For this purpose, STAT2 levels were analyzed in absence or presence of the proteasome inhibitor MG132. As shown in Figure 3E, STAT2 levels were partially restored in the presence of MG132 inhibitor in pI215L expressing cells. This result supports the hypothesis of pI215L being the viral factor responsible for STAT2 degradation through the proteasome. This role was confirmed by immunoprecipitation assays in COS-1 cells transfected with EV or pIRES-I215L-myc plasmids. As depicted in Figure 3F, the ubiquitination pattern of STAT2 in I215L transfected cells was more intense compared to EV-control cells. Based on that, we speculate that pI215L specifically induced the ubiquitination of STAT2 before its degradation by the proteasome, thus controlling the inhibition of the JAK/STAT signaling pathway.

### The inhibition of IFN-I-dependent genes depends on the ubiquitin-conjugating activity of pI215L

**Figure 4:**
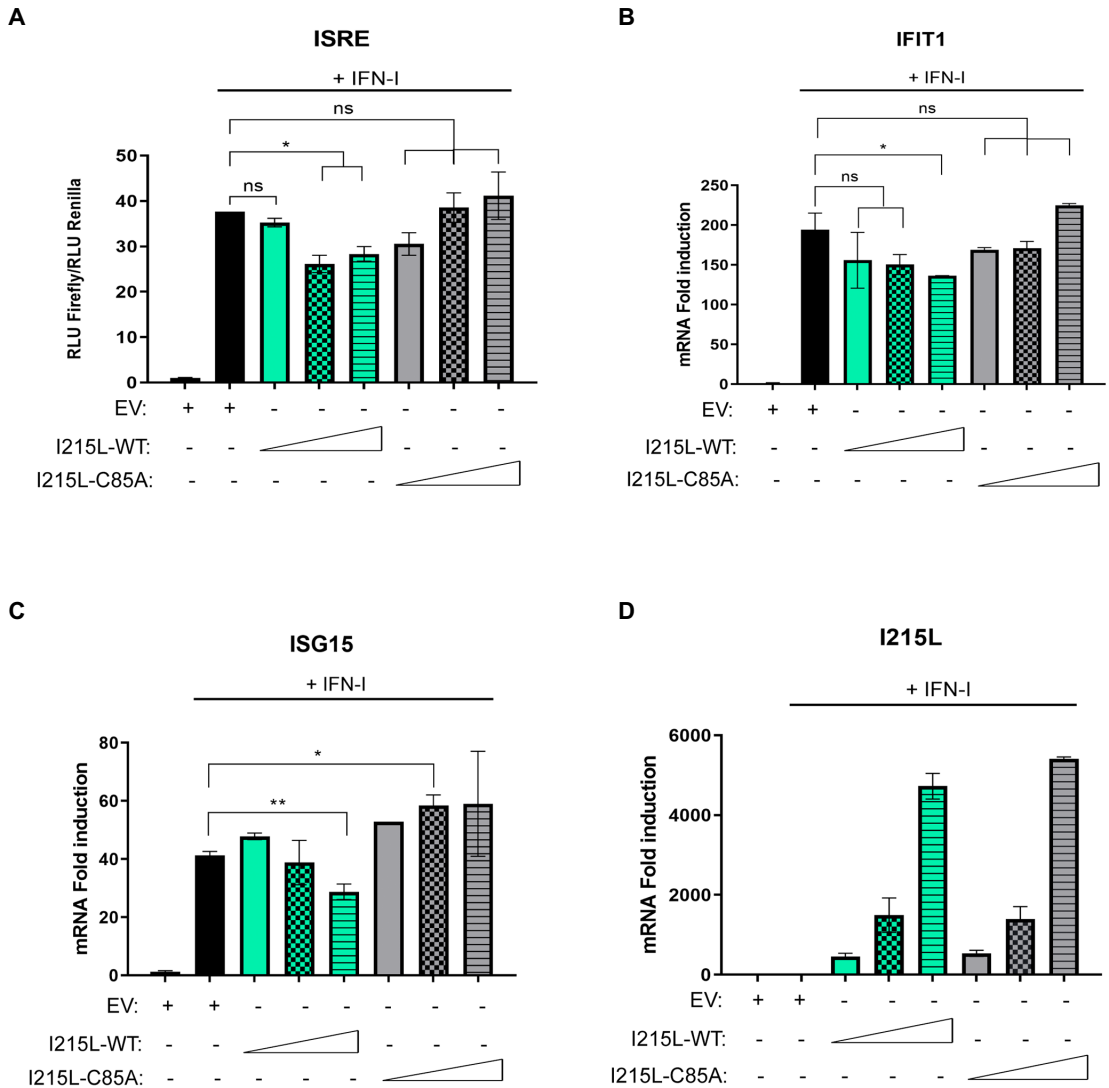
pI215L-dependent inhibition of IFN-I-mediated signaling pathway relies on its ubiquitin-conjugating activity. **(A)** ISRE-luciferase reporter experiment was performed in HEK-293 cells transfected with EV or with increasing concentrations of either pIRES-I215L-WT-myc or pIRES-I215L-C85A-myc (0.3, 1, or 3 μg/1 × 10^6^ cells) in absence or in presence of IFN-I (500 U/mL). Graph represents the means of the Firefly luciferase RLU (Relative Luminiscence Units) values divided by its Renilla luciferase RLU values from biological triplicates. Obtained values were relativized against the untreated EV transfected sample. **(B)** IFIT1 and **(C)** ISG15 mRNA levels were analyzed in COS-1 cells transfected with EV (2.5 μg/1 × 10^6^ cells) or increasing concentrations of pIRES-I215L-WT-myc or pIRES-I215L-C85A-myc (0.25, 1, or 2.5 μg/1 × 10^6^ cells) by qRT-PCR. **(D)** As a control of I215L transfection, I215L mRNA levels were also amplified by qRT-PCR in the same conditions than A-C. All data reflect mean ± SEM (*n* = 3). Data were statistically analyzed by using a Student t test (*p < 0.05; **p < 0.01; ns: not significant).

It has been described that the viral protein I215L modulates several signaling pathways of the innate immune response independently of its ubiquitin-conjugating activity, such as inhibiting NFκB and AP1 activation (Barrado-Gil et al., 2021) or counteracting the cGAS-STING signaling pathway by inhibiting TBK1 activation (Huang et al., 2021). Thus, we were interested to clarify whether the ubiquitin conjugating activity of pI215L is required or not for the inhibition of the IFN-I signaling pathway. To assess this, we investigated the functional consequences of the pI215L mutation at its described catalytic domain (Cys85Ala; Freitas et al., 2018), on the expression of the IFN-I-dependent genes. For this purpose, we first analyzed ISRE activity by luciferase assay (Figure 4A) in HEK-293 cells transfected with EV or with increasing concentrations of either pIRES-I215L-wild type (WT)-myc or the mutant pIRES-I215L-C85A-myc, treated or not with IFN-I. Whereas expression of increasing concentrations of I215L-WT resulted in the inhibition of IFN-I-stimulated ISRE promoter activity, increasing concentrations of I215L-C85A did not inhibit its activity, but rather showed a slight dose-dependent increasing tendency, suggesting the importance of the E2-ubiquitin-conjugating activity in the control of the pathway by this viral protein. To corroborate that, we monitored the mRNA induction of the ISGs IFIT1 (Figure 4B) and ISG15 (Figure 4C) by qPCR in COS-1 cells, transfected with increasing concentrations of I215L-WT versus similar amounts of I215L-C85A. The mRNA induction of both IFIT1 and ISG15 by IFN-I was inhibited only in the presence of increasing doses of I215L-WT, whereas it was not significantly diminished in the presence of I215L-C85A. As control, the mRNA level of both WT and C85A I215L were assessed (Figure 4D).

These data clearly indicate that the inhibition of the JAK/STAT pathway by I215L strongly depends on its catalytic domain, and thus, on its E2 activity.

### Ubiquitin-conjugating function is not needed for STAT2 interaction but is required for its ubiquitination and degradation

**Figure 5:**
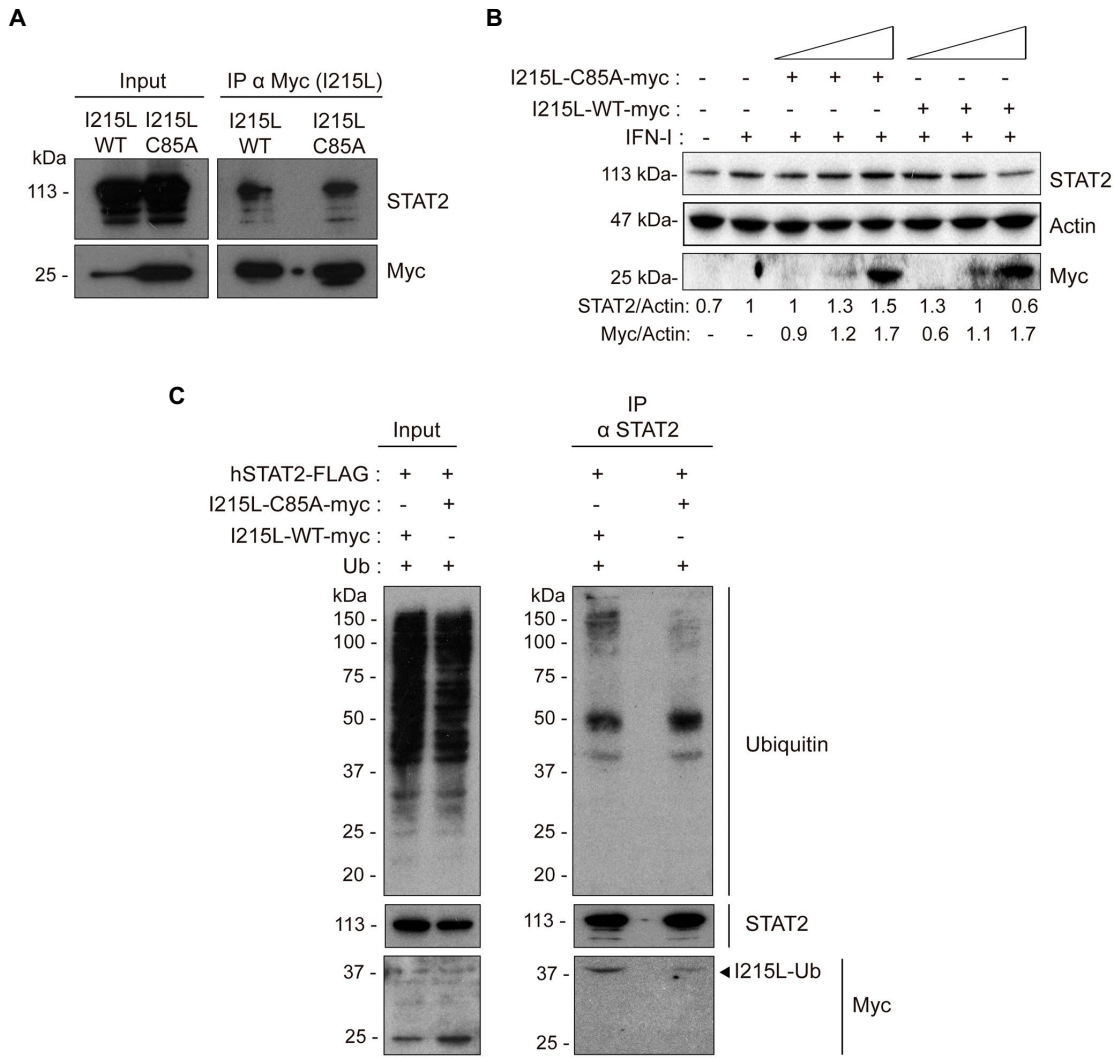
The ubiquitin-conjugating catalytic domain of I215L is not required for interaction with STAT2 but is required for its ubiquitination and degradation. **(A)** Immunoprecipitation of I215L-WT or I215L-C85A in BSRT7 cells co-transfected with hSTAT2-FLAG and with either pIRES-I215L-WT-myc or pIRES-I215L-C85A-myc, using an anti-myc antibody. BSRT7 cells were co-transfected with hSTAT2-FLAG (0.4 μg/1 × 10^6^ cells) and either pIRES-I215L-WT-myc or pIRES-I215L-C85A-myc (2 μg/1 × 10^6^ cells) plasmids. At 24 h post-transfection, cells were treated with IFN-I (500 U/mL) for 1 h. Then, cells were collected and lysed for immunoprecipitation assay with A/G magnetic beads using anti-myc antibody. Western blot labeling anti-STAT2, anti-myc and anti-actin antibodies is shown. **(B)** STAT2 levels were analyzed in presence of increasing concentrations of either I215L-C85A-myc or I215L-WT-myc protein (0.25, 1, or 2.5 μg/1 × 10^6^ cells) or EV (2.5 μg/1 × 10^6^ cells) in HEK-293 by Western blot assay. Antibodies against STAT2, myc and actin were used. STAT2 and pI215L-myc levels were quantified according with their actin levels and relativized with the EV control using ImageJ. **(C)** Immunoprecipitation of STAT2 in Vero cells transfected with either pIRES-I215L-WT-myc or pIRES-I215L-C85A-myc. Vero cells were co-transfected with pCI-His-hUbiquitin (1 μg/1 × 10^6^ cells), hSTAT2-FLAG (0.4 μg/1 × 10^6^ cells) and either pIRES-I215L-WT-myc or pIRES-I215L-C85A-myc (2 μg/1 × 10^6^ cells) plasmids. At 24 h post-transfection, cells were treated with IFN-I (500 U/mL) for 2 h. Cells were collected and lysed for STAT2 immunoprecipitation assay with A/G magnetic beads. Western blot labeling with anti-ubiquitin, anti-STAT2, anti-myc, and anti-actin antibodies is shown.

As the ubiquitin-conjugating activity has been found to be essential for the control of the pathway, as described above, we next analyzed whether the interaction of pI215L with STAT2 would depend on its catalytic activity. To achieve this purpose, BSRT7 cells were co-transfected with hSTAT2-FLAG, and either with pIRES-I215L-WT-myc or pIRES-I215L-C85A-myc, harvested, and processed for immunoprecipitation. Western blot confirmed the interaction of I215L-WT with hSTAT2-FLAG, and interestingly, showed that I215L-C85A also interacts with hSTAT2-FLAG (Figure 5A). As a negative control for immunoprecipitation, cells were transfected with empty vector as shown in Figure 2A.

On the other hand, and to further assess the functional mechanism of the viral protein, we analyzed the involvement of the catalytic site of pI215L on STAT2 degradation in HEK-293 cells (Figure 5B). As shown in Figure 5B, and as expected, the expression of increasing doses of I215L-WT decreased STAT2 levels, whereas expression of I215L-C85A proportionally increased STAT2 levels. To corroborate that the specific STAT2 degradation by ASFV pI215L is mediated by the ubiquitin conjugation and subsequent proteasomal degradation, an analysis of STAT2 ubiquitination was assessed in Vero cells transfected with I215L-WT or I215L-C85A followed by an immunoprecipitation assay. The immunoprecipitated hSTAT2-FLAG showed a much higher ubiquitination pattern in the I215L-WT-containing sample, compared to the sample expressing I215L-C85A (Figure 5C), which showed a ubiquitination pattern similar to the observed in the EV-transfected samples (Figure 3F). As can be seen in the WB panel labeled with anti-myc antibody of Figure 5C, both pI215L-WT-myc and pI215L-C85A-myc co-immunoprecipitated with hSTAT2-FLAG, detected at a molecular weight of 37 kDa, corresponding with the binding of one molecule of ubiquitin to pI215L (pI215L-Ub). Differences in the amount of co-immunoprecipitated pI215L-Ub from pI215L-WT-myc or pI215L-C85A transfected samples, would be expected as the mutation on the catalytic domain would affect its ubiquitin binding capacity and its conjugation (Freitas et al., 2018), which is consistent with the differences in ubiquitination patterns of hSTAT2-FLAG. These data are in line with those showed at the Figure 5B, in which STAT2 degradation is observed only if pI215L exerts its ubiquitin-conjugating function.

Therefore, all together these data evidence that the antagonism of the IFN-I pathway carried out by ASFV pI215L is due to STAT2 degradation, further requiring its E2-ubiquitin-conjugating enzyme function for such degradation, but not for the interaction with STAT2.

### ASFV infection induces STAT2 proteasomal degradation through its interaction with pI215L

**Figure 6:**
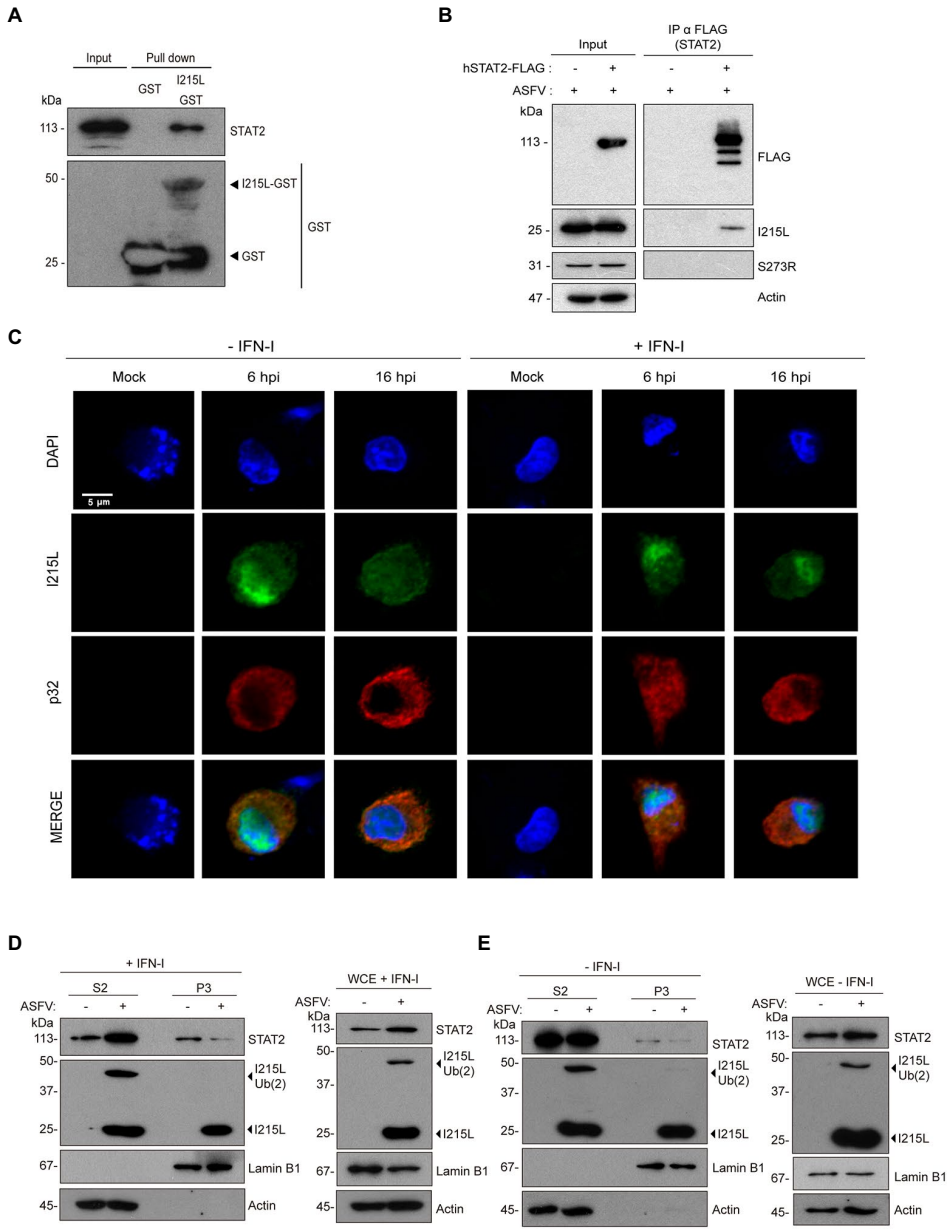
pI215L interacts with STAT2 and promotes its ubiquitination during infection in PAMs. **(A)** pI215L-GST interacts with STAT2 from extracts of infected PAMs by a pull-down assay. PAMs were infected with Arm/07/CBM/c2 (1 PFU/cell) harvested at 16 hpi and processed for the pull-down experiment with glutathione sepharose beads bound to pI215L-GST or to GST alone as a negative control. Bead-bound proteins were detected by Western blotting labeling with anti-STAT2 and anti-GST antibodies. **(B)** hSTAT2-FLAG-pI215L interaction during ASFV infection. COS-1 cells were transfected with hSTAT2-FLAG (0.4 μg/1 × 10^6^ cells) and infected with Arm/07/CBM/c2 (1 PFU/cell) for 16 h. Cells were then collected and processed for immunoprecipitation with an anti-FLAG antibody and analyzed by Western blot analysis labeling with antibodies against FLAG to detect STAT2, against pI215L, against pS273R (negative control of co-inmunoprecipitation) and against actin. **(C)** PAMs were mock infected or infected with Armenia/07/CBM/c2 (1 PFU/cell). At 5 or 15 hpi, cells were untreated or treated with universal type I IFN (250 U/mL). After 1 h of treatment, cells were fixed and stained with DAPI (blue), anti-pI215L (green) and anti-p32 (red) antibodies and examined by a confocal microscope. Individual and merged images of the different channels are shown. **(D,E)** Nuclear fractionation of mock-infected or infected PAMs at 16 hpi in presence **(D)** or in absence **(E)** of IFN-I (500 U/mL). PAMs were seeded in p60 plates and were mock infected (−) or infected (+) with Armenia/07/CBM/c2 strain (1 PFU/cell) for 16 h. At 15 hpi, cells were untreated or treated with type I IFN (500 U/mL) for 1 h. Then, cells were collected and nuclear fractionation was performed. The whole cell extract (WCE), cytoplasmic fraction (S2) and nuclear chromatin fraction (P3) were analyzed by 10% SDS-PAGE, followed by immunoblotting with anti-STAT2 and anti-I215L antibodies. As controls of the fractionation, antibody against nuclear lamin B1 and antibody against cytoplasmic actin were used.

To corroborate the function of pI215L in the context of ASFV infection, we used the ASFV strain Arm/07/CBM/c2 (Gallardo et al., 2015; Pérez-Núñez et al., 2020) to perform a pull down in infected PAMs, aiming to show the interaction of endogenous STAT2 with I215L-GST in these conditions, as explained in Materials and Methods section (Figure 6A). The experiment corroborated the interaction of pI215L-GST with endogenous STAT2, obtained from Arm/07/CBM/c2-infected PAMs, whereas the negative GST control did not bind endogenous STAT2. To further confirm this finding, COS-1 cells were first transfected with hSTAT2-FLAG or EV and then infected with Arm/07/CBM/c2 for 16 h (Figure 6B). STAT2 immunoprecipitation was performed, and samples were analyzed by Western blot. Importantly, STAT2-pI215L interaction was clearly detected, confirming the data during ASFV infection in COS-1 cells. Additionally, as further control of the specificity of the STAT2-I215L interaction, we analyzed the co-immunoprecipitation of STAT2 with another ASFV protein, the VPPA protease (S273R), confirming that it did not interact with STAT2, thus reinforcing the specificity of pI215L-STAT2 interaction in the context of infection (Figure 6B). Finally, we have confirmed pI215L-STAT2 colocalization in the nuclear compartment during Arm/07/CBM/c2 infection in PAM at 6 and 16 hpi (Supplementary Figure 4).

Since we have described above that pI215L and STAT2 colocalize in the nucleus both in ectopic expression experiments and during ASFV infection (Figures 2, 5, 6A, B; Supplementary Figure 4), and in order to describe the distribution of pI215L during infection, we analyzed its location in Arm/07/CBM/c2-infected PAMs, either in the presence or absence of IFN-I, at 6 or 16 hpi, by immunofluorescence (Figure 6C). At 6 hpi, pI215L localized into the nucleus both in the absence and in presence of IFN-I (97% or 100% of infected cells, respectively), suggesting a virus-induced mechanism to bring the viral protein to the nucleus, independent of IFN I stimulation. At 16 hpi, the percentage of IFN-I unstimulated cells expressing pI215L in the nucleus goes down, and only the 42% showed a nuclear pattern. In addition, 16 h infected-cells stimulated with IFN-I revealed a pI215L nuclear pattern in 61% of the total number of infected cells (Supplementary Figure 5A). These results revealed significant differences in the nuclear location pattern of pI215L at 16 hpi between unstimulated and IFN-I-stimulated samples, while these differences were not significant at 6 hpi. The cytoplasmic distribution of pI215L detected by immunofluorescence at 16 hpi both in the absence (58%) and presence of IFN-I (39%) showed either a diffuse cytoplasmic pattern or concentrated in viral factories detected with DAPI (Supplementary Figure 5B). Thus, these data would indicate that pI215L goes to the nucleus at early stages of infection, mainly after the activation of the JAK/STAT pathway, to inhibit the STAT2-dependent transcription of ISGs, and this late nuclear localization is enhanced by the presence of IFN-I.

Finally, to confirm the role of pI215L on STAT2 ubiquitination and degradation during ASFV infection, a nucleus-cytoplasm fractionation was further performed. For this purpose, mock-infected or infected PAMs with Arm/07/CBM/c2 (1 PFU/cell) were either stimulated (Figure 6D) or not (Figure 6E) with IFN-I at 15 hpi. In the cytoplasmic fraction (S2), no significant differences in STAT2 levels were observed between mock and infected macrophages in absence of IFN-I, highlighting the importance of IFN-I in the virus-mediated regulation of STAT2. Thus, in IFN-I-stimulated cells, STAT2 levels in the cytoplasm were higher during the infection compared to the mock samples, whereas in the nuclear chromatin-binding fraction (P3), a marked decrease of STAT2 was observed. In this regard, while pI215L was detected both in its non-ubiquitinated (I215L, 25 kDa) and ubiquitin-conjugated conformations (I215L-Ub (2), 47 kDa) either in the WCE or in the cytoplasmic fraction, only the non-ubiquitinated conformation was detected in the chromatin-bound fraction. These data suggest that pI215L may conjugate ubiquitin at the nucleus, thus promoting STAT2 degradation and explaining the drop in nuclear STAT2 levels, which would represent the mechanism, or one of the mechanisms, by which the virus control the IFN-I signaling pathway and the regulation of the promoters of the IFN I-dependent genes.

## Discussion

The ability of ASFV to counteract the activation of the innate response is critical to prevent the establishment of the antiviral immune response. Probably for this reason, ASFV encodes several genes devoted to the control of IFN-I, mainly within the MGF360 and MGF505/530 multigene families, aiming to evade host defenses (Afonso et al., 2004; Correia et al., 2013; O’Donnell et al., 2015; Reis et al., 2016). Not only that, but many other ASFV genes not belonging to these multigene families have also been involved in the modulation of the immune response, such as A238L, which inhibits the expression of TNF-alpha, the transcriptional co-activator p300 or iNOS (Granja et al., 2006a,b; Granja et al., 2008, 2009); I329L protein that impair the activation of IFN-β and CCL5 by inhibiting TLR3 (de Oliveira et al., 2011) or the proteins DP96R (Wang et al., 2018), S273R (Luo et al., 2022) and E120R (Liu et al., 2021), among others, which counteract IFN-I production by inhibiting cGAS-STING pathway.

In addition to that, the degree of virulence during the infection with different ASFV viral strains has been related to the capacity of the virus to inhibit cell signaling pathways that trigger the establishment of the innate antiviral response. Our laboratory has described that modulation of IFN-beta production through the cGAS-STING signaling pathway differs between virulent and attenuated isolates, being inhibited by virulent, but not by the attenuated ASFV strains (García-Belmonte et al., 2019). Recently, we have also described that both the virulent Arm/07/CBM/c2 and attenuated NH/P68 ASFV strains inhibit the IFN-I signaling pathway, both through the activation of the proteasomal degradation of STAT2 and by caspase 3-dependent cleavage of STAT1 (Riera et al., 2021).

As mentioned above, while many ASFV genes have been described to be involved in the modulation of the IFN-I production by inhibiting the cGAS-STING pathway (reviewed in (García-Belmonte et al., 2022)), there are few studies focused on the identification of the factor(s) involved in JAK/STAT pathway modulation, which constitutes the second central transduction pathway mediating IFN anti-viral responses. Among the ASFV genes involved in JAK/STAT pathway modulation, MGF505-7R was described as a negative regulator of both type I and type II IFN signaling pathways by using reporter luciferase assays in Vero cells (Correia et al., 2013) and, recently, its mechanism of action has been identified in ectopic experiments, suggesting that MGF505-7R interacts and induces the degradation of JAK1 and JAK2 (D. Li et al., 2021). Moreover, it has been reported that the protein codified by MGF360-9 l gene inhibits IFN-β signaling by both STAT1 degradation through apoptosis and by STAT2 proteasomal degradation (Zhang et al., 2022).

The ubiquitin-conjugating ASFV protein I215L has recently been described to impair IFN-I production preventing the activation of cGAS-STING pathway through the inhibition of TBK1 phosphorylation and activation (Huang et al., 2021). pI215L also impairs IFN-I production by blocking the activation of NF-kB and AP-1 transcription factors (Barrado-Gil et al., 2021), demonstrated in ectopic experiments. Additionally, pI215L has been reported to interfere with the cellular gene transcription (Bulimo et al., 2000), and with the translation and proteasomal machinery by interacting with the cap-dependent translation machinery initiation factor eIF4E, the 40S ribosomal protein RPS23 and the E3 ligase Cullin 4B (Cul4B; Barrado-Gil et al., 2021).

In this study, we have characterized a molecular mechanism by which pI215L inhibits the activation of the IFN-I signaling pathway not only ectopically but more importantly, during ASFV infection. Moreover, we have related for the first time the E2-ubiquitin-conjugating activity of pI215L with its inhibitory activity over IFN-I signaling pathway. Ectopic expression of pI215L inhibits ISRE promoter activity and mRNA induction of the IFN-I-stimulated genes ISG15 and IFIT1 upon IFN-I stimulation, being this inhibition pI215L-dose-dependent and strictly relies on the E2 activity. In line with this, a recently published paper by Li et al. (2022) showed that pI215L inhibited IFN-I signaling by degrading IRF9, implying the I215L protein as a modulator of the JAK/STAT pathway. However, the intrinsic pI215L E2-ubiquitin-conjugating function was not shown to be involved in the inhibition of the IFN-I pathway. In this regard, we show here that whereas the pI215L-STAT2 interaction does not depend on the catalytic activity of pI215L, STAT2 degradation does. These data also indicate that pI215L-STAT2 interaction occurs in another domain of the I215L protein, excluding the catalytic domain in the interaction. Hence, the ubiquitin-conjugating activity has been proved to be fundamental for STAT2 degradation.

Our data determine that the ASFV ubiquitin E2-conjugating enzyme pI215L interacts and colocalizes with STAT2 in the nuclear compartment to promote its degradation, and this interaction appears to occur when STAT2 forms the STAT1/STAT2 heterodimer of the ISGF3 complex, since STAT1 also coimmunoprecipitates with pI215L. Interestingly, pI215 has been described to interact with Cul4B (Barrado-Gil et al., 2021) in this cellular compartment. In addition, pI215L-induced STAT2 degradation compromise STAT2 activation since a reduction of STAT2 phosphorylation is also observed, suggesting that phosphorylation of STAT2 is affected due to the depletion of global STAT2 during pI215L-WT expression. The reduction of endogenous STAT2 levels caused by pI215L-WT expression was found to be stronger in human HEK293 cells than in COS-1 and Vero monkey cells. This would be explained by the fact that STAT2 is the most divergent component of the JAK/STAT pathway (Blaszczyk et al., 2015), so there might be different efficiencies in the recognition and degradation of STAT2 by pI215L, depending on STAT2 species.

Importantly, we have described that STAT2 degradation by pI215L is proteasome-dependent, since we have seen that pI215L induces its ubiquitination and, besides, that STAT2 levels are restored in the presence of the proteasome inhibitor MG132 in pI215L expressing samples. Importantly, our studies carried out during ASFV infection confirmed the early localization of pI215L in the nucleus, both in the absence and in the presence of IFN-I, and the relationship between the presence of IFN-I and the maintenance of nuclear localization of pI215L. The early location of pI215L in the nuclear compartment during ASFV infection in absence of IFN-I stimulation could be explained by the fact that an initial IFN-I induction is observed at early times of infection (García-Belmonte et al., 2019), thus pI215L may block the IFN-I-induced signaling in the nucleus. In support of this, the nucleus-cytoplasm fractionation experiments revealed that pI215L would induce the downregulation of STAT2 levels in the nuclear compartment. Furthermore, it could be speculated that pI215L is conjugating ubiquitin at the nuclear compartment, probably to degrade STAT2.

It is known that STAT2 is the key element negatively regulated by viruses regarding IFN-I signaling, whereas it is likely that the participation of STAT1 is not that necessary, especially in the non-canonical IFN-I signaling as reported in Blaszczyk et al. (2015) and Hahm et al. (2005). More specifically, several viruses carry out STAT2 degradation through the expression of viral proteins that interfere with the cellular proteasomal system, such as the NS5 protein of the flaviviruses DENV (Ashour et al., 2009; Morrison et al., 2013) and ZIKV (Grant et al., 2016) or paramyxovirus V protein (Ramachandran and Horvath, 2009). On the other hand, herpes simplex virus 2 (HSV2) encodes ICP22 protein, an E3 ubiquitin ligase that ubiquitinates and degrades STAT1, STAT2 and IRF9 *via* the proteasomal pathway (Kadeppagari et al., 2012; Zhang et al., 2020), and cytomegaloviruses encode pUL145 and M27, which degrade STAT2 by using DDB1-Culin4A/B-Roc1 proteasome mechanism (Döring et al., 2014).

**Figure 7:**
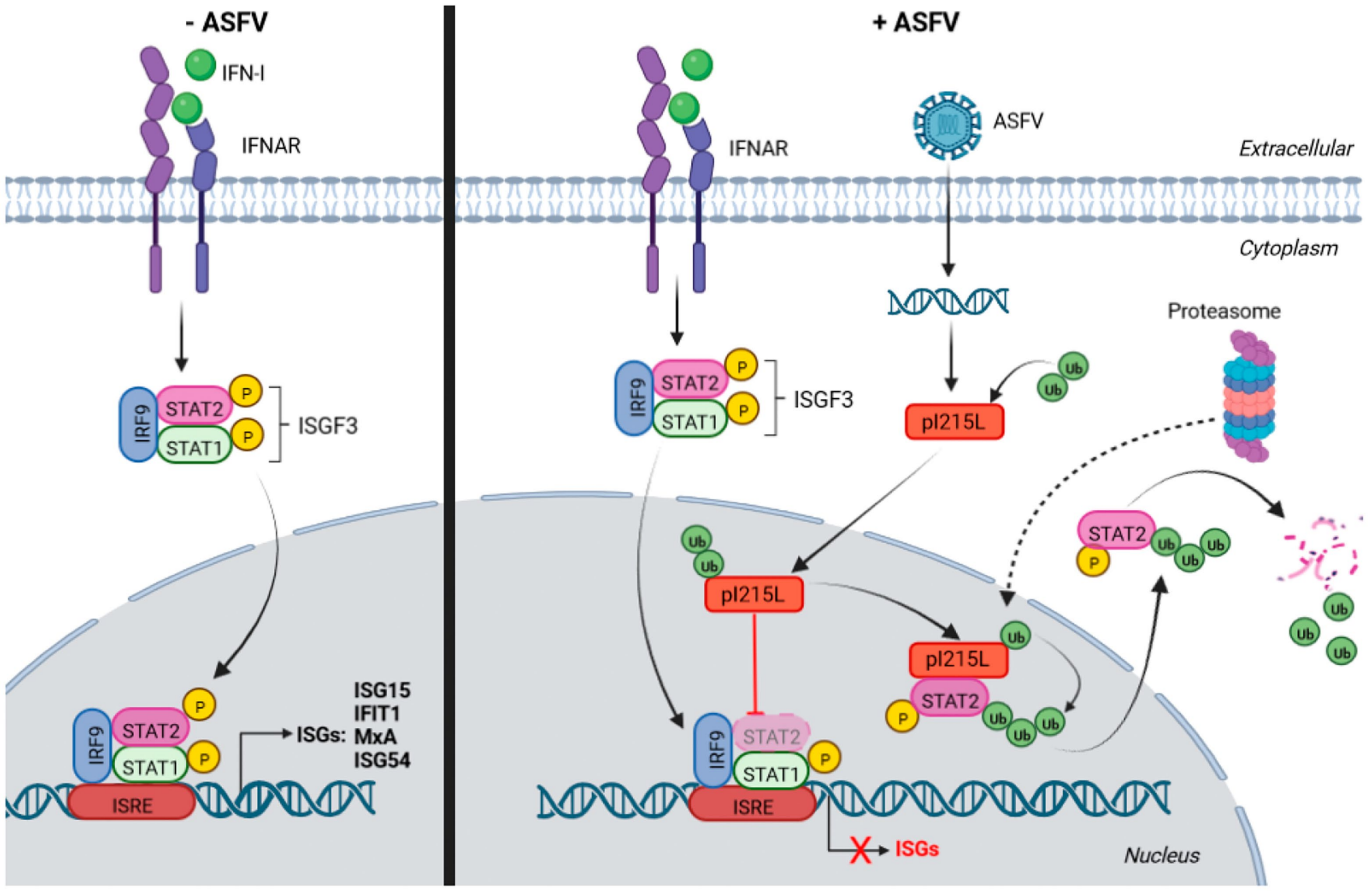
Schematic representation of the model of action of pI215L inhibiting the JAK/STAT pathway *via* STAT2 degradation. On the right side, the activation of the JAK/STAT signaling pathway upon IFN-I stimulus in a non-infected cell is depicted: STAT1 and STAT2 are phosphorylated, together with IRF9 conform the ISGF3 complex, which translocates to the nucleus to act as a transcription factor to induce ISGs transcription. The right side shows the activation of the JAK/STAT pathway in an ASFV-infected cell, where the viral protein I215L interacts with STAT2 in the nucleus, promoting its ubiquitination and degradation, thus preventing ISGF3 complex formation and therefore the transcription of the genes involved in the response to IFN-I. STAT2 degradation by the proteasome has been depicted in the cytoplasm, although the hypothesis that the proteasomal machinery translocates into the nucleus to carry out the degradation of ubiquitinated STAT2 (dashed arrow), cannot be excluded. Biorender program was used for the realization of this illustration.

In conclusion, this study provides the first evidence on the role of the catalytic activity of the E2-ubiquitin-conjugating enzyme pI215L on the modulation of the innate immune response, specifically through the JAK/STAT signaling pathway. Based on these data, we proposed a model schematized in Figure 7, in which pI215L translocates into the nucleus at early stages of infection to interact with STAT2 and to promote its ubiquitination and subsequent proteasomal degradation. Future studies have to be carried out to identify the cellular factors of the proteasomal machinery involved in pI215L-induced STAT2 degradation, which may contribute in the future to the generation of tools for next generation-ASFV vaccine prototypes.

## Data availability statement

The original contributions presented in the study are included in the article/Supplementary material, further inquiries can be directed to the corresponding author.

## Author contributions

ER: design of the work, analysis, and interpretation of data for the work and drafting of the first working draft. RG-B: acquisition, analysis and interpretation of data. RM: drafting the work. DP-N: drafting the work and revising it critically for important intellectual content. YR: drafting the work, revising it critically for important intellectual content, and final approval of the version to be published. All authors contributed to the article and approved the submitted version.

## Funding

This work has been supported by grant IND2018/BIO-9398 from Autonomous Community of Madrid (Spain). Within Industrial PhD grant, ER was economically supported by BioAssays company.

## Conflict of interest

RM was employed by Bioassays SL.

The remaining authors declare that the research was conducted in the absence of any commercial or financial relationships that could be construed as a potential conflict of interest.

The authors declare that this study received funding from BioAssays company. The funder had the following involvement in the study: drafting the work and revising it critically for important intellectual content.

## Publisher’s note

All claims expressed in this article are solely those of the authors and do not necessarily represent those of their affiliated organizations, or those of the publisher, the editors and the reviewers. Any product that may be evaluated in this article, or claim that may be made by its manufacturer, is not guaranteed or endorsed by the publisher.

## Supplementary material

SUPPLEMENTARY FIGURE 1 I215L gene encodes for an early protein. PAMs were infected with Arm/07/CBM/c2 ASFV strain (2 PFU/cell) in absence or in presence of AraC (40 mg/ml). Cells were collected at 24 hpi, lysed in RIPA buffer and analyzed by Western blot. Antibodies against ASFV-pI215L, p72 (ASFV late protein), p32 (ASFV early protein) and actin were used. pI215L levels were quantified according with their actin levels and relativized to the 4 hpi sample from Figure 1B by using ImageJ.

SUPPLEMENTARY FIGURE 2 pI215L co-interacts with hSTAT1-FLAG from STAT1/STAT2 complex in the nucleus. BSRT7 cells were co-transfected with hSTAT1-FLAG (0.4 μg/1x106 cells) and with pIRES-I215L-myc (2 µg/1x106 cells). 24 hours post-transfection, cells were stimulated with Universal Type I IFN (500 U/ml) for 1h. Cells were then collected and processed for immunoprecipitation with an anti-myc antibody and analyzed by Western blot labelling with antibodies against FLAG to detect STAT1, against myc to detect pI215L and against actin.

SUPPLEMENTARY FIGURE 3 pI215L promotes STAT2 degradation. STAT2 levels were analyzed in presence of increasing concentrations of pIRES-I215L-myc (0.25, 1 or 2.5 µg/1x106 cells) or EV (2.5 µg/1x106 cells) in COS-1 **(A)** or Vero **(B)** cells by Western blot assay. Antibodies against STAT2, myc and actin were employed. STAT2 levels were quantified according with their actin levels and relativized with the EV control using ImageJ and are indicated below the panels (n=3).

SUPPLEMENTARY FIGURE 4 pI215L colocalizes with STAT2 in the nucleus during Arm/07/CBM/c2 infection in PAMs. PAMs were mock infected or infected with Armenia/07/CBM/c2 (1 PFU/cell). At 5 or 15 hpi, cells were treated with universal type I IFN (250 U/ml). After 1 h of treatment, cells were fixed and stained with DAPI (blue), anti-STAT2 (green) and anti-pI215L (red) antibodies and examined by a confocal microscope. Individual and merged images of the different channels are shown. For improved detection of STAT2 and pI215L colocalization, a MERGE image of both channels (green and red) was also generated.

SUPPLEMENTARY FIGURE 5 Nuclear localization of pI215L occurs from early stages of infection and decrease at late stage of infection, but is maintained in the presence of IFN-I. PAMs were mock infected or infected with Armenia/07/CBM/c2 (1 PFU/cell). At 5 or 15 hpi, cells were untreated or treated with universal type I IFN (250 U/ml). After 1 h of treatment, cells were fixed and stained with DAPI, anti-pI215L and anti-p32 antibodies and examined by a confocal microscope. **(A)** The number of cells expressing pI215L in the nuclear compartment was counted and the percentage of pI215L-positive cells in the nucleus was obtained based on the total number of infected cells. Data is represented as the Mean ± SEM. More than 50 cells were counted per condition using different fields (n≥3) from two independent experiments. A Two Way ANOVA with multiple comparison analysis with Bonferroni post-tests was performed, as shown in the Figure (****p* < 0.001; ns: not significant). **(B)** PAMs were mock infected or infected with Armenia/07/CBM/c2 (1 PFU/cell). At 5 or 15 hpi, cells were untreated or treated with universal type I IFN (250 U/ml). After 1 h of treatment, cells were fixed and stained with DAPI (blue) and anti-pI215L (green) antibodies and examined by a confocal microscope. Individual and merged images of the different channels are shown.

## References

[ref1] Afonso C. L. Piccone M. E. Zaffuto K. M. Neilan J. Kutish G. F. Lu Z.. (2004). African swine fever virus multigene family 360 and 530 genes affect host interferon response. J. Virol. 78, 1858–1864. doi: 10.1128/jvi.78.4.1858-1864.2004, PMID: 14747550 PMC369441

[ref2] Ashour J. Laurent-Rolle M. Shi P.-Y. García-Sastre A. (2009). NS5 of dengue virus mediates STAT2 binding and degradation. J. Virol. 83, 5408–5418. doi: 10.1128/jvi.02188-08, PMID: 19279106 PMC2681973

[ref3] Banjara S. Caria S. Dixon L. K. Hinds M. G. Kvansakul M. (2017). Structural insight into African swine fever virus A179L-mediated inhibition of apoptosis. J. Virol. 91:e02228-16. doi: 10.1128/jvi.02228-16, PMID: 28053104 PMC5331815

[ref4] Barrado-Gil L. Del Puerto A. Galindo I. Cuesta-Geijo M. Á. García-Dorival I. de Motes C. M.. (2021). African swine fever virus ubiquitin-conjugating enzyme is an Immunomodulator targeting NF-κB activation. Viruses 13:1160. doi: 10.3390/V13061160, PMID: 34204411 PMC8233900

[ref5] Blaszczyk K. Olejnik A. Nowicka H. Ozgyin L. Chen Y. L. Chmielewski S.. (2015). STAT2/IRF9 directs a prolonged ISGF3-like transcriptional response and antiviral activity in the absence of STAT1. Biochem. J. 466, 511–524. doi: 10.1042/BJ20140644, PMID: 25564224 PMC4403947

[ref6] Bulimo W. D. Miskin J. E. Dixon L. K. (2000). An ARID family protein binds to the African swine fever virus encoded ubiquitin conjugating enzyme, UBCv1. FEBS Lett. 471, 17–22. doi: 10.1016/S0014-5793(00)01352-1, PMID: 10760505

[ref01] Carrascosa A. L. Santarén J. F. Viñuela E. (1982). Production and titration of African swine fever virus in porcine alveolar macrophages. J. Virol. Methods 3, 303–310. doi: 10.1016/0166-0934(82)90034-9 7085838

[ref7] Correia S. Ventura S. Parkhouse R. M. (2013). Identification and utility of innate immune system evasion mechanisms of ASFV. Virus Res. 173, 87–100. doi: 10.1016/j.virusres.2012.10.013, PMID: 23165138

[ref8] Darnell J. E. Kerr I. M. Stark G. R. (1994). Jak-STAT pathways and transcriptional activation in response to IFNs and other extracellular signaling proteins. Science 264, 1415–1421. doi: 10.1126/science.8197455, PMID: 8197455

[ref9] de Oliveira V. L. Almeida S. C. P. Soares H. R. Crespo A. Marshall-Clarke S. Parkhouse R. M. E. (2011). A novel TLR3 inhibitor encoded by African swine fever virus (ASFV). Arch. Virol. 156, 597–609. doi: 10.1007/S00705-010-0894-7/FIGURES/6, PMID: 21203785 PMC3066390

[ref10] Dixon L. K. Chapman D. A. G. Netherton C. L. Upton C. (2013). African swine fever virus replication and genomics. Virus Res. 173, 3–14. doi: 10.1016/j.virusres.2012.10.020 23142553

[ref11] Dixon L. K. Stahl K. Jori F. Vial L. Pfeiffer D. U. (2020). African swine fever epidemiology and control. Annu. Rev. Anim. Biosci. 8, 221–246. doi: 10.1146/annurev-animal-021419 31743062

[ref12] Döring M. Lessin I. Frenz T. Spanier J. Kessler A. Tegtmeyer P.. (2014). M27 expressed by cytomegalovirus counteracts effective type I interferon induction of myeloid cells but not of Plasmacytoid dendritic cells. J. Virol. 88, 13638–13650. doi: 10.1128/JVI.00216-14, PMID: 25231302 PMC4248974

[ref13] Elliott J. Lynch O. T. Suessmuth Y. Qian P. Boyd C. R. Burrows J. F.. (2007). Respiratory syncytial virus NS1 protein degrades STAT2 by using the Elongin-Cullin E3 ligase. J. Virol. 81, 3428–3436. doi: 10.1128/jvi.02303-06, PMID: 17251292 PMC1866062

[ref14] Freitas F. B. Frouco G. Martins C. Ferreira F. (2018). African swine fever virus encodes for an E2-ubiquitin conjugating enzyme that is mono-and di-ubiquitinated and required for viral replication cycle. Sci. Rep. 8:3471. doi: 10.1038/s41598-018-21872-2, PMID: 29472632 PMC5823848

[ref15] Gallardo C. Nieto R. Soler A. Pelayo V. Fernández-Pinero J. Markowska-Daniel I.. (2015). Assessment of African swine fever diagnostic techniques as a response to the epidemic outbreaks in eastern european union countries: how to improve surveillance and control programs. J. Clin. Microbiol. 53, 2555–2565. doi: 10.1128/JCM.00857-15, PMID: 26041901 PMC4508403

[ref16] García-Belmonte R. Pérez-Núñez D. Pittau M. Richt J. A. Revilla Y. (2019). African swine fever virus Armenia/07 virulent strain controls interferon beta production through the cGAS-STING pathway. J. Virol. 93:e02298-18. doi: 10.1128/jvi.02298-18, PMID: 30918080 PMC6613762

[ref17] García-Belmonte R. Pérez-Núñez D. Revilla Y. (2022). Controlling the cGAS-STING pathway: the signature of ASFV virulence. J. Immunol. Sci. 6, 1–5. doi: 10.29245/2578-3009/2022/2.1233

[ref18] Granja A. G. Nogal M. L. Hurtado C. del Aguila C. Carrascosa A. L. Salas M. L.. (2006a). The viral protein A238L inhibits TNF-α expression through a CBP/p300 transcriptional Coactivators pathway. J. Immunol. 176, 451–462. doi: 10.4049/jimmunol.176.1.451, PMID: 16365438

[ref19] Granja A. G. Perkins N. D. Revilla Y. (2008). A238L inhibits NF-ATc2, NF-κB, and c-Jun activation through a novel mechanism involving protein kinase C-θ-mediated up-regulation of the amino-terminal transactivation domain of p300. J. Immunol. 180, 2429–2442. doi: 10.4049/jimmunol.180.4.2429 18250452

[ref20] Granja A. G. Sabina P. Salas M. L. Fresno M. Revilla Y. (2006b). Regulation of inducible nitric oxide synthase expression by viral A238L-mediated inhibition of p65/RelA acetylation and p300 transactivation. J. Virol. 80, 10487–10496. doi: 10.1128/jvi.00862-06, PMID: 17041221 PMC1641776

[ref21] Granja A. G. Sánchez E. G. Sabina P. Fresno M. Revilla Y. (2009). African swine fever virus blocks the host cell antiviral inflammatory response through a direct inhibition of PKC-θ-mediated p300 transactivation. J. Virol. 83, 969–980. doi: 10.1128/jvi.01663-08, PMID: 19004945 PMC2612362

[ref22] Grant A. Ponia S. S. Tripathi S. Balasubramaniam V. Miorin L. Sourisseau M.. (2016). Zika virus targets human STAT2 to inhibit type i interferon signaling. Cell Host Microbe. 19, 882–890. doi: 10.1016/j.chom.2016.05.009, PMID: 27212660 PMC4900918

[ref23] Hahm B. Trifilo M. J. Zuniga E. I. Oldstone M. B. A. (2005). Viruses evade the immune system through type I interferon-mediated STAT2-dependent, but STAT1-independent, Signaling. Immunity 22, 247–257. doi: 10.1016/J.IMMUNI.2005.01.005, PMID: 15723812

[ref24] Hernáez B. Díaz-Gil G. García-Gallo M. Quetglas J. I. Rodríguez-Crespo I. Dixon L.. (2004). The African swine fever virus dynein-binding protein p54 induces infected cell apoptosis. FEBS Lett. 569, 224–228. doi: 10.1016/j.febslet.2004.06.001, PMID: 15225638

[ref25] Hingamp P. M. Arnold J. E. Mayer R. J. Dixon L. K. (1992). A ubiquitin conjugating enzyme encoded by African swine fever virus. EMBO J. 11, 361–366. doi: 10.1002/J.1460-2075.1992.TB05058.X, PMID: 1310934 PMC556456

[ref26] Hingamp P. M. Leyland M. L. Webb J. Twigger S. Mayer R. J. Dixon L. K. (1995). Characterization of a ubiquitinated protein which is externally located in African swine fever virions. J. Virol. 69, 1785–1793. doi: 10.1128/JVI.69.3.1785-1793.1995, PMID: 7853518 PMC188786

[ref27] Huang L. Xu W. Liu H. Xue M. Liu X. Zhang K.. (2021). African swine fever virus pI215L negatively regulates cGAS-STING signaling pathway through recruiting RNF138 to inhibit K63-linked Ubiquitination of TBK1. J. Immunol. 207, 2754–2769. doi: 10.4049/JIMMUNOL.2100320 34759016

[ref28] Huangfu W.-C. Fuchs S. Y. (2010). Ubiquitination-dependent regulation of signaling receptors in cancer. Genes Cancer 1, 725–734. doi: 10.1177/1947601910382901, PMID: 21127735 PMC2994580

[ref29] Kadeppagari R. K. Sanchez R. L. Foster T. P. (2012). HSV-2 inhibits type-I interferon signaling via multiple complementary and compensatory STAT2-associated mechanisms. Virus Res. 167, 273–284. doi: 10.1016/J.VIRUSRES.2012.05.010, PMID: 22634037 PMC3398234

[ref30] Kawai T. Akira S. (2006). Innate immune recognition of viral infection. Nat. Immunol. 7, 131–137. doi: 10.1038/ni1303 16424890

[ref31] Kim T. K. Maniatis T. (1996). Regulation of interferon-γ-activated STAT1 by the ubiquitin-proteasome pathway. Science 273, 1717–1719. doi: 10.1126/SCIENCE.273.5282.1717, PMID: 8781235

[ref32] Li L. Fu J. Li J. Guo S. Chen Q. Zhang Y.. (2022). African swine fever virus pI215L inhibits type I interferon signaling by targeting interferon regulatory factor 9 for Autophagic degradation. J. Virol. 96:e0094422. doi: 10.1128/JVI.00944-22, PMID: 35972295 PMC9472647

[ref33] Li D. Zhang J. Yang W. Li P. Ru Y. Kang W.. (2021). African swine fever virus protein MGF-505-7R promotes virulence and pathogenesis by inhibiting JAK1-and JAK2-mediated signaling. J. Biol. Chem. 297:101190. doi: 10.1016/J.JBC.2021.101190, PMID: 34517008 PMC8526981

[ref34] Liu H. Zhu Z. Feng T. Ma Z. Xue Q. Wu P.. (2021). African swine fever virus E120R protein inhibits interferon Beta production by interacting with IRF3 to block its activation. J. Virol. 95, 824–845. doi: 10.1128/JVI.00824-21/SUPPL_FILE/JVI.00824-21-S0004.XLSX PMC8387055 34190598

[ref35] Lo M. S. Brazas R. M. Holtzman M. J. (2005). Respiratory syncytial virus nonstructural proteins NS1 and NS2 mediate inhibition of Stat2 expression and alpha/beta interferon responsiveness. J. Virol. 79, 9315–9319. doi: 10.1128/JVI.79.14.9315-9319.2005, PMID: 15994826 PMC1168759

[ref36] Luo J. Zhang J. Ni J. Jiang S. Xia N. Guo Y.. (2022). The African swine fever virus protease pS273R inhibits DNA sensing cGAS-STING pathway by targeting IKKε. Virulence 13, 740–756. doi: 10.1080/21505594.2022.2065962, PMID: 35437104 PMC9067533

[ref37] Lurie R. H. Platanias L. C. (2005). Mechanisms of type-I- and type-II-interferon-mediated signalling. Nat. Rev. Immunol 5, 375–386. doi: 10.1038/nri1604 15864272

[ref02] Méndez J. Stillman B. (2000). Chromatin association of human origin recognition complex, Cdc6, and minichromosome maintenance proteins during the cell cycle: assembly of prereplication complexes in late mitosis. Mol. Cell. Biol. 20, 8602–8612. doi: 10.1128/mcb.20.22.8602-8612.2000 11046155 PMC102165

[ref38] Meyer T. Vinkemeier U. (2004). Nucleocytoplasmic shuttling of STAT transcription factors. Eur. J. Biochem. 271, 4606–4612. doi: 10.1111/j.1432-1033.2004.04423.x 15606747

[ref03] Miorin L. Laurent-Rolle M. Pisanelli G. Hendrick Co P. Albrecht R. A. García-Sastre A.. (2019). Host-Specific NS5 ubiquitination determines yellow fever virus tropism. J. Virol. 93:e00151-19. doi: 10.1128/JVI.00151-19 31043530 PMC6600188

[ref39] Morrison J. Laurent-Rolle M. Maestre A. M. Rajsbaum R. Pisanelli G. Simon V.. (2013). Dengue virus co-opts UBR4 to degrade STAT2 and antagonize type I interferon signaling. PLoS Pathog. 9:e1003265. doi: 10.1371/journal.ppat.1003265, PMID: 23555265 PMC3610674

[ref40] O’Donnell V. Holinka L. G. Gladue D. P. Sanford B. Krug P. W. Lu X.. (2015). African swine fever virus Georgia isolate harboring deletions of MGF360 and MGF505 genes is attenuated in swine and confers protection against challenge with virulent parental virus. J. Virol. 89, 6048–6056. doi: 10.1128/JVI.00554-15, PMID: 25810553 PMC4442422

[ref41] Pérez-Núñez D. Castillo-Rosa E. Vigara-Astillero G. García-Belmonte R. Gallardo C. Revilla Y. (2020). Identification and isolation of two different subpopulations within african swine fever virus arm/07 stock. Vaccine 8:625. doi: 10.3390/vaccines8040625, PMID: 33113838 PMC7712101

[ref42] Ramachandran A. Horvath C. M. (2009). Paramyxovirus disruption of interferon signal transduction: STATus report. J. Interf. Cytokine Res. 29, 531–537. doi: 10.1089/JIR.2009.0070, PMID: 19694544 PMC2956620

[ref04] Ramos R. Martínez-Salas E. (1999). Long-range RNA interactions between structural domains of the aphthovirus internal ribosome entry site (IRES). RNA. 5, 1374–83. doi: 10.1017/s1355838299991240 10573128 PMC1369859

[ref43] Reis A. L. Abrams C. C. Goatley L. C. Netherton C. Chapman D. G. Sanchez-Cordon P.. (2016). Deletion of African swine fever virus interferon inhibitors from the genome of a virulent isolate reduces virulence in domestic pigs and induces a protective response. Vaccine 34, 4698–4705. doi: 10.1016/j.vaccine.2016.08.011, PMID: 27521231 PMC5012891

[ref44] Revilla Y. Cebrián A. Baixerás E. Martínez-A C. Viñuela E. Salas M. L. (1997). Inhibition of apoptosis by the African swine fever virus Bcl-2 homologue: role of the BH1 domain. Virology 228, 400–404. doi: 10.1006/viro.1996.8395, PMID: 9123849

[ref45] Riera E. Pérez-Núñez D. García-Belmonte R. Miorin L. García-Sastre A. Revilla Y. (2021). African swine fever virus induces STAT1 and STAT2 degradation to counteract IFN-I signaling. Front. Microbiol. 12:722952. doi: 10.3389/FMICB.2021.722952, PMID: 34512601 PMC8427279

[ref46] Rodríguez J. M. Salas M. L. (2013). African swine fever virus transcription. Virus Res. 173, 15–28. doi: 10.1016/j.virusres.2012.09.014 23041356

[ref47] Rodriguez J. M. Salas M. L. Viñuela E. (1992). Genes homologous to ubiquitin-conjugating proteins and eukaryotic transcription factor SII in African swine fever virus. Virology 186, 40–52. doi: 10.1016/0042-6822(92)90059-X, PMID: 1309282

[ref48] Sánchez-Cordón P. J. Montoya M. Reis A. L. Dixon L. K. (2018). African swine fever: A re-emerging viral disease threatening the global pig industry. Vet. J. 233, 41–48. doi: 10.1016/J.TVJL.2017.12.025, PMID: 29486878 PMC5844645

[ref49] Shuai K. Liu B. (2003). Regulation of JAK–STAT signalling in the immune system. Nat. Rev. Immunol. 3, 900–911. doi: 10.1038/nri1226 14668806

[ref50] Stark G. R. Darnell J. E. (2012). The JAK-STAT pathway at twenty. Immunity. 36, 503–514. doi: 10.1016/j.immuni.2012.03.013, PMID: 22520844 PMC3909993

[ref51] Thompson M. R. Kaminski J. J. Kurt-Jones E. A. Fitzgerald K. A. (2011). Pattern recognition receptors and the innate immune response to viral infection. Viruses 3, 920–940. doi: 10.3390/v3060920, PMID: 21994762 PMC3186011

[ref52] Trilling M. Le V. T. K. Fiedler M. Zimmermann A. Bleifuß E. Hengel H. (2011). Identification of DNA-damage DNA-binding protein 1 as a conditional essential factor for cytomegalovirus replication in interferon-γ-stimulated cells. PLoS Pathog. 7:e1002069. doi: 10.1371/JOURNAL.PPAT.1002069, PMID: 21698215 PMC3116810

[ref53] Ulane C. M. Horvath C. M. (2002). Paramyxoviruses SV5 and HPIV2 assemble STAT protein ubiquitin ligase complexes from cellular components. Virology 304, 160–166. doi: 10.1006/viro.2002.1773, PMID: 12504558

[ref54] Ulane C. M. Kentsis A. Cruz C. D. Parisien J.-P. Schneider K. L. Horvath C. M. (2005). Composition and assembly of STAT-targeting ubiquitin ligase complexes: paramyxovirus V protein carboxyl terminus is an oligomerization domain. J. Virol. 79, 10180–10189. doi: 10.1128/JVI.79.16.10180-10189.2005, PMID: 16051811 PMC1182666

[ref55] Viswanathan K. Früh K. DeFilippis V. (2010). Viral hijacking of the host ubiquitin system to evade interferon responses. Curr. Opin. Microbiol. 13, 517–523. doi: 10.1016/J.MIB.2010.05.012, PMID: 20699190 PMC2939720

[ref56] Wang X. Wu J. Wu Y. Chen H. Zhang S. Li J.. (2018). Inhibition of cGAS-STING-TBK1 signaling pathway by DP96R of ASFV China 2018/1. Biochem. Biophys. Res. Commun. 506, 437–443. doi: 10.1016/J.BBRC.2018.10.103, PMID: 30348523

[ref57] Yáñez R. J. Rodríguez J. M. Nogal M. L. Yuste L. Enríquez C. Rodriguez J. F.. (1995). Analysis of the complete nucleotide sequence of African swine fever virus. Virology 208, 249–278. doi: 10.1006/viro.1995.1149 11831707

[ref58] Zhang M. Fu M. Li M. Hu H. Gong S. Hu Q. (2020). Herpes simplex virus type 2 inhibits type I IFN signaling mediated by the novel E3 ubiquitin protein ligase activity of viral protein ICP22. J. Immunol. 205, 1281–1292. doi: 10.4049/JIMMUNOL.2000418 32699158

[ref59] Zhang K. Yang B. Shen C. Zhang T. Hao Y. Zhang D.. (2022). MGF360-9L is a major virulence factor associated with the African swine fever virus by antagonizing the JAK/STAT signaling pathway. MBio 13:e0233021. doi: 10.1128/MBIO.02330-21, PMID: 35076286 PMC8788333

[ref60] Zimmermann A. Trilling M. Wagner M. Wilborn M. Bubic I. Jonjic S.. (2005). A cytomegaloviral protein reveals a dual role for STAT2 in IFN-{gamma} signaling and antiviral responses. J. Exp. Med. 201, 1543–1553. doi: 10.1084/JEM.20041401, PMID: 15883169 PMC2212917

[ref61] Zuo Y. Feng Q. Jin L. Huang F. Miao Y. Liu J.. (2020). Regulation of the linear ubiquitination of STAT1 controls antiviral interferon signaling. Nat. Commun. 11, 1146–1115. doi: 10.1038/s41467-020-14948-z, PMID: 32123171 PMC7052135

