# Supplementary figures and images for: African swine fever virus ubiquitin-conjugating enzyme pI215L inhibits IFN-I signaling pathway through STAT2 degradation
Source: Front Microbiol. 2023 Jan 13;13:1081035. doi: 10.3389/fmicb.2022.1081035 (PMC9880986; doi:10.3389/fmicb.2022.1081035)

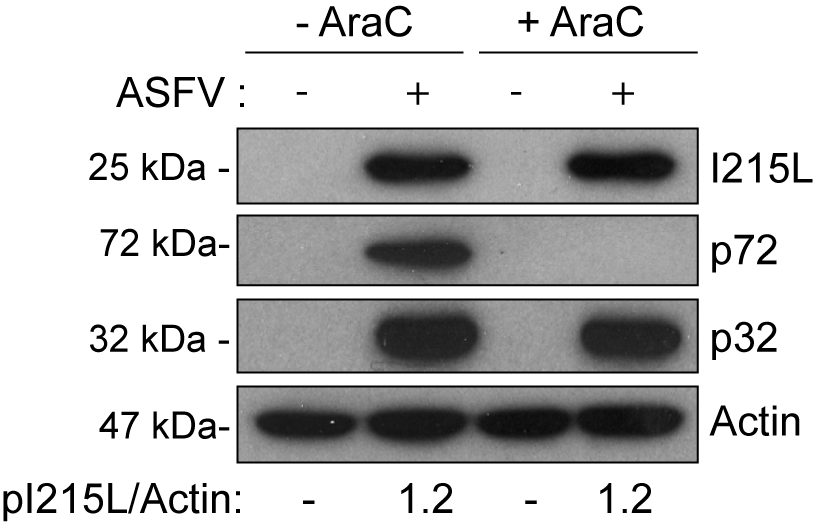

Supplement: SUPPLEMENTARY FIGURE 1 — I215L gene encodes for an early protein. PAMs were infected with Arm/07/CBM/c2 ASFV strain (2 PFU/cell) in absence or in presence of AraC (40 mg/ml). Cells were collected at 24 hpi, lysed in RIPA buffer and analyzed by Western blot. Antibodies against ASFV-pI215L, p72 (ASFV late protein), p32 (ASFV early protein) and actin were used. pI215L levels were quantified according with their actin levels and relativized to the 4 hpi sample from Figure 1B by using ImageJ. [file Image_1.TIF]

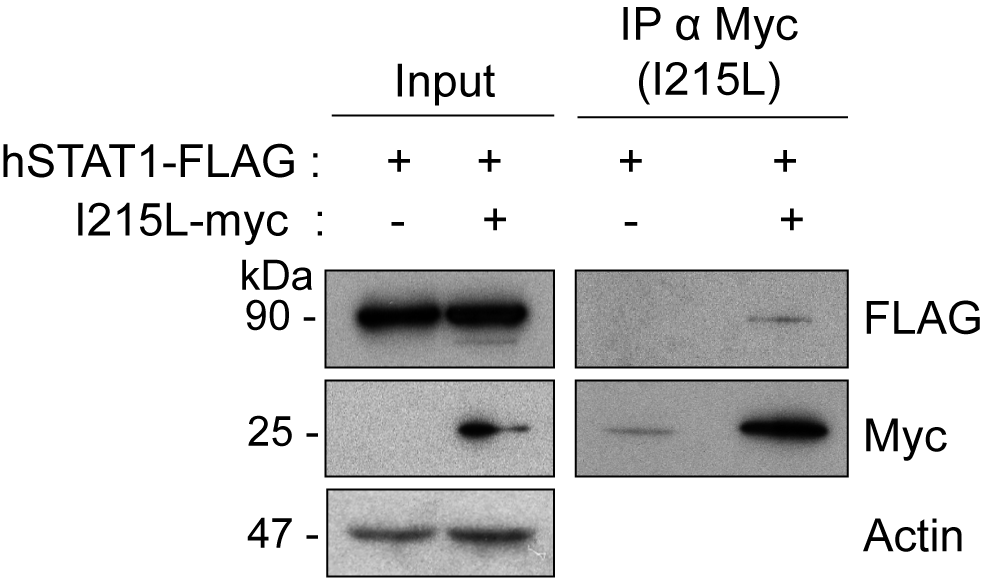

Supplement: SUPPLEMENTARY FIGURE 2 — pI215L co-interacts with hSTAT1-FLAG from STAT1/STAT2 complex in the nucleus. BSRT7 cells were co-transfected with hSTAT1-FLAG (0.4 μg/1x106 cells) and with pIRES-I215L-myc (2 µg/1x106 cells). 24 hours post-transfection, cells were stimulated with Universal Type I IFN (500 U/ml) for 1h. Cells were then collected and processed for immunoprecipitation with an anti-myc antibody and analyzed by Western blot labelling with antibodies against FLAG to detect STAT1, against myc to detect pI215L and against actin. [file Image_2.TIF]

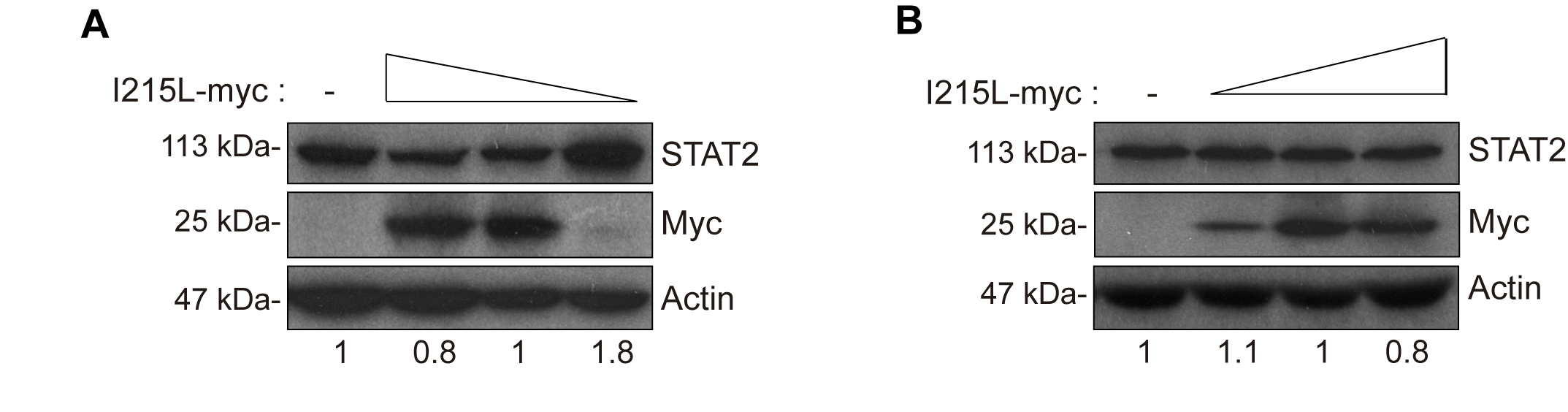

Supplement: SUPPLEMENTARY FIGURE 3 — pI215L promotes STAT2 degradation. STAT2 levels were analyzed in presence of increasing concentrations of pIRES-I215L-myc (0.25, 1 or 2.5 µg/1x106 cells) or EV (2.5 µg/1x106 cells) in COS-1 (A) or Vero (B) cells by Western blot assay. Antibodies against STAT2, myc and actin were employed. STAT2 levels were quantified according with their actin levels and relativized with the EV control using ImageJ and are indicated below the panels (n=3). [file Image_3.TIF]

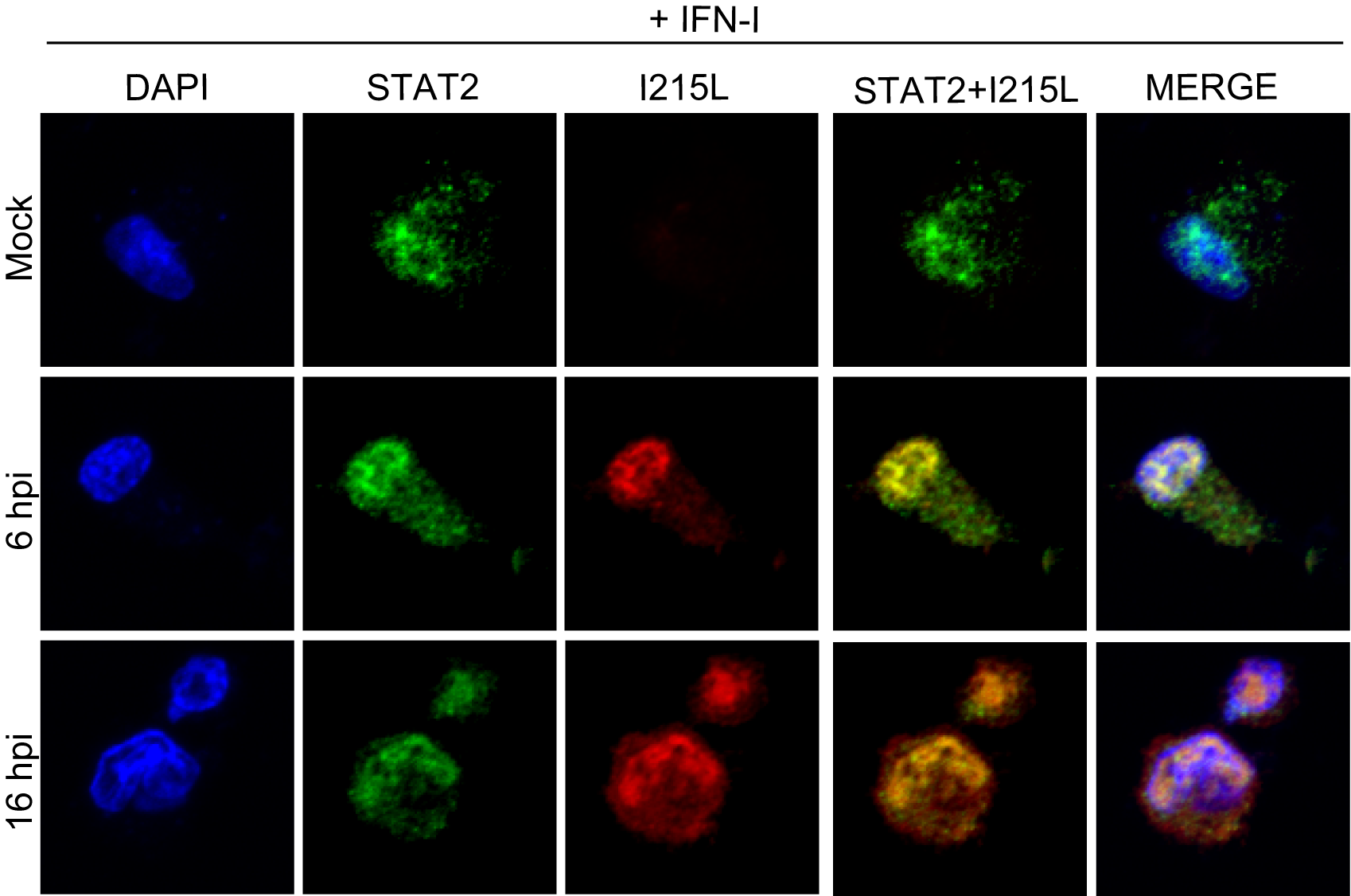

Supplement: SUPPLEMENTARY FIGURE 4 — pI215L colocalizes with STAT2 in the nucleus during Arm/07/CBM/c2 infection in PAMs. PAMs were mock infected or infected with Armenia/07/CBM/c2 (1 PFU/cell). At 5 or 15 hpi, cells were treated with universal type I IFN (250 U/ml). After 1 h of treatment, cells were fixed and stained with DAPI (blue), anti-STAT2 (green) and anti-pI215L (red) antibodies and examined by a confocal microscope. Individual and merged images of the different channels are shown. For improved detection of STAT2 and pI215L colocalization, a MERGE image of both channels (green and red) was also generated. [file Image_4.TIF]

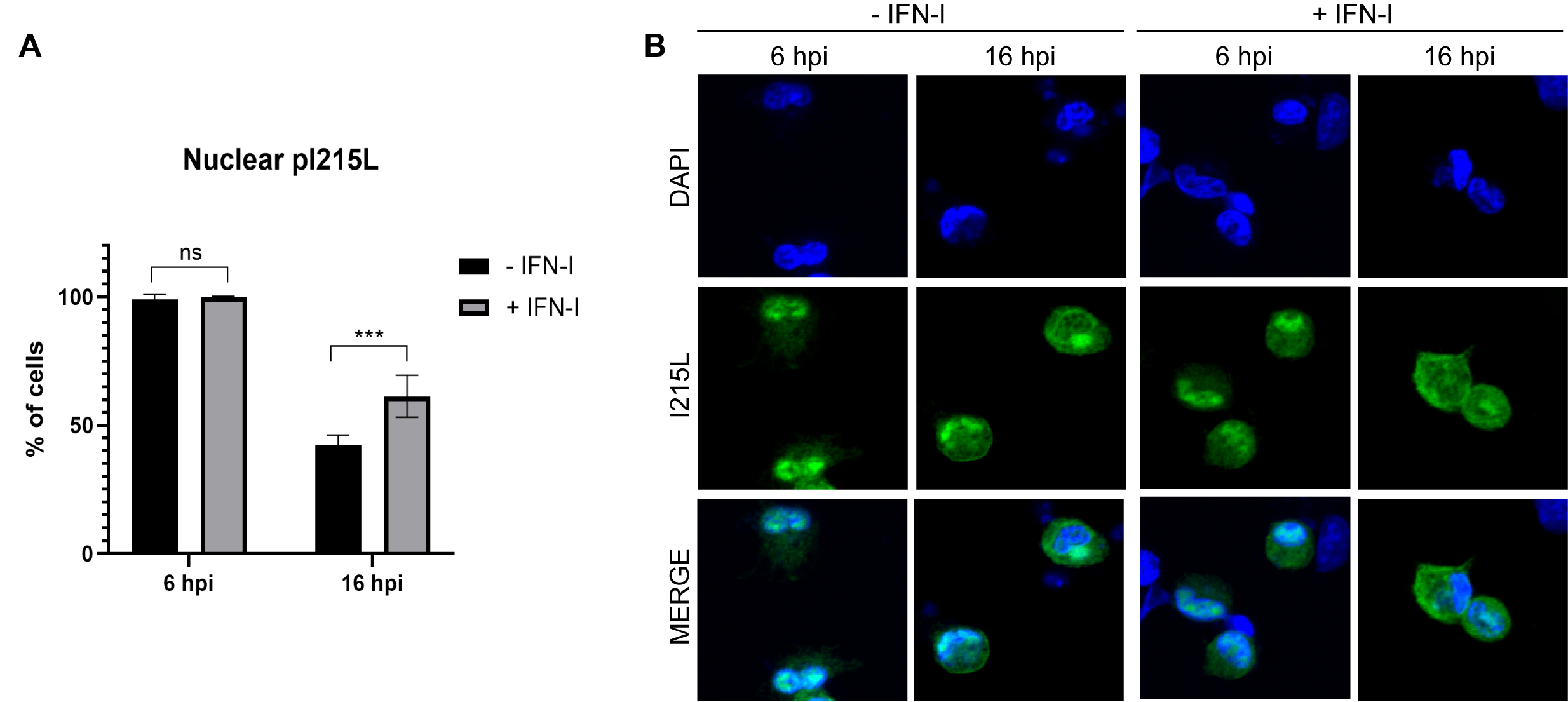

Supplement: SUPPLEMENTARY FIGURE 5 — Nuclear localization of pI215L occurs from early stages of infection and decrease at late stage of infection, but is maintained in the presence of IFN-I. PAMs were mock infected or infected with Armenia/07/CBM/c2 (1 PFU/cell). At 5 or 15 hpi, cells were untreated or treated with universal type I IFN (250 U/ml). After 1 h of treatment, cells were fixed and stained with DAPI, anti-pI215L and anti-p32 antibodies and examined by a confocal microscope. (A) The number of cells expressing pI215L in the nuclear compartment was counted and the percentage of pI215L-positive cells in the nucleus was obtained based on the total number of infected cells. Data is represented as the Mean ± SEM. More than 50 cells were counted per condition using different fields (n≥3) from two independent experiments. A Two Way ANOVA with multiple comparison analysis with Bonferroni post-tests was performed, as shown in the Figure (***p < 0.001; ns: not significant). (B) PAMs were mock infected or infected with Armenia/07/CBM/c2 (1 PFU/cell). At 5 or 15 hpi, cells were untreated or treated with universal type I IFN (250 U/ml). After 1 h of treatment, cells were fixed and stained with DAPI (blue) and anti-pI215L (green) antibodies and examined by a confocal microscope. Individual and merged images of the different channels are shown. [file Image_5.TIF]
